# Novel Protein Biomarkers and Therapeutic Targets for Type 1 Diabetes and Its Complications: Insights from Summary-Data-Based Mendelian Randomization and Colocalization Analysis

**DOI:** 10.3390/ph17060766

**Published:** 2024-06-11

**Authors:** Mingrui Zou, Jichun Yang

**Affiliations:** 1 Department of Physiology and Pathophysiology, School of Basic Medical Sciences, Key Laboratory of Molecular Cardiovascular Science of the Ministry of Education, Center for Non-Coding RNA Medicine, Peking University Health Science Center, Beijing 100191, China; 2 Peking University First School of Clinical Medicine, Peking University First Hospital, Beijing 100034, China; 2110301145@stu.pku.edu.cn

**Keywords:** GWAS, Mendelian randomization, plasma proteins, therapeutic targets, type 1 diabetes

## Abstract

Millions of patients suffer from type 1 diabetes (T1D) and its associated complications. Nevertheless, the pursuit of a cure for T1D has encountered significant challenges, with a crucial impediment being the lack of biomarkers that can accurately predict the progression of T1D and reliable therapeutic targets for T1D. Hence, there is an urgent need to discover novel protein biomarkers and therapeutic targets, which holds promise for targeted therapy for T1D. In this study, we extracted summary-level data on 4907 plasma proteins from 35,559 Icelanders and 2923 plasma proteins from 54,219 UK participants as exposures. The genome-wide association study (GWAS) summary statistics on T1D and T1D with complications were obtained from the R9 release results from the FinnGen consortium. Summary-data-based Mendelian randomization (SMR) analysis was employed to evaluate the causal associations between the genetically predicted levels of plasma proteins and T1D-associated outcomes. Colocalization analysis was utilized to investigate the shared genetic variants between the exposure and outcome. Moreover, transcriptome analysis and a protein–protein interaction (PPI) network further illustrated the expression patterns of the identified protein targets and their interactions with the established targets of T1D. Finally, a Mendelian randomization phenome-wide association study evaluated the potential side effects of the identified core protein targets. In the primary SMR analysis, we identified 72 potential protein targets for T1D and its complications, and nine of them were considered crucial protein targets. Within the group were five risk targets and four protective targets. Backed by evidence from the colocalization analysis, the protein targets were classified into four tiers, with MANSC4, CTRB1, SIGLEC5 and MST1 being categorized as tier 1 targets. Delving into the DrugBank database, we retrieved 11 existing medications for T1D along with their therapeutic targets. The PPI network clarified the interactions among the identified potential protein targets and established ones. Finally, the Mendelian randomization phenome-wide association study corroborated MANSC4 as a reliable target capable of mitigating the risk of various forms of diabetes, and it revealed the absence of adverse effects linked to CTRB1, SIGLEC5 and MST1. This study unveiled many protein biomarkers and therapeutic targets for T1D and its complications. Such advancements hold great promise for the progression of drug development and targeted therapy for T1D.

## 1. Introduction

Type 1 diabetes (T1D), a prevalent chronic autoimmune disease, is characterized by the dysfunction of islet β-cells, leading to reduced insulin levels and elevated blood glucose levels [1]. This form of diabetes predominantly affects adolescents, with its peak incidence observed at 12–14 years of age [2]. According to statistics from Green et al., in 2017, the estimated prevalent and incident cases of T1D were 9,004,610 and 234,710, respectively [3]. The 10th edition of the International Diabetes Federation (IDF) Atlas estimated that 1,211,900 adolescents younger than 20 years old had T1D globally [4]. Moreover, the complications of T1D pose significant challenges for patients, predominantly manifesting as microvascular complications (neuropathy, retinopathy and nephropathy) and macrovascular complications (cerebrovascular disease, coronary artery disease and peripheral arterial disease) [1]. A study conducted at the University of Dundee in Scotland revealed that T1D and its complications diminished the life expectancy of individuals by 11 years [5]. However, a cure for T1D remains elusive at present, with a significant challenge being the lack of reliable biomarkers that can accurately predict the progression of T1D and reliable therapeutic targets for T1D [6,7].

Plasma proteins are vital components of human blood, playing crucial roles in molecular transport and signal transduction, as well as tissue growth and repair [8]. Additionally, the levels of plasma proteins provide an insight into the human health state, thereby holding promise as biomarkers and therapeutic targets for diseases [9]. This potential avenue offers new insights for the early diagnosis, prognosis assessment and targeted treatment of diseases. At present, some studies have attempted to integrate bioinformatics analysis with case–control studies to identify plasma proteins as promising biomarkers for T1D [6,10,11]. For example, Nakayasu et al. screened 83 biomarkers related to T1D through case–control studies. In addition, they also used machine learning algorithms to validate the diagnostic performance of these biomarkers [6]. However, there are still significant limitations. Firstly, conventional observational studies are unable to avoid the interference of confounding factors and reverse causal bias or to establish the causal associations between biomarkers and T1D. Furthermore, conducting research on multiple diseases simultaneously is difficult. Therefore, traditional approaches cannot guarantee the specificity of the identified targets. Therefore, a reliable analytical approach is imperative to overcome the deficiencies in the current research.

Mendelian randomization (MR) analysis stands as an epidemiological strategy that leverages genetic variants as instrumental variables (IVs) to investigate the causal associations between exposure and outcomes [12]. Notably, MR analysis is less susceptible to confounding factors and reverse causal bias, owing to the random allocation of genetic variants at conception [13]. There are three fundamental assumptions of MR analysis: (1) the correlation assumption—the genetic IVs exhibit robust correlations with the exposure; (2) the independence assumption—the IVs remain unaffected by confounding factors linked to the exposure or outcome; (3) the exclusivity assumption—the IVs are exclusively allowed to influence the outcome via the exposure [12]. With the availability of data from large-scale proteomic studies, the protein quantitative trait loci (pQTLs) of plasma proteins can be utilized to illuminate the causal effects of plasma protein levels on diseases through MR analysis, which enables us to identify novel protein biomarkers and therapeutic targets for diseases [14,15]. Moreover, a novel form of MR analysis known as summary-data-based Mendelian randomization (SMR) analysis has emerged, which employs the top associated cis-QTL as the IV. This approach boasts significantly enhanced statistical power compared to traditional MR analysis [16,17]. To date, proteome-wide MR and SMR analysis have provided significant insights into novel biomarkers of diseases such as inflammatory bowel disease, osteoarthritis and colorectal cancer [17,18,19]. However, no research has yet employed MR analysis to investigate the causal associations between plasma proteins and T1D along with its associated complications, with the aim of unveiling novel therapeutic targets.

**Figure 1:**
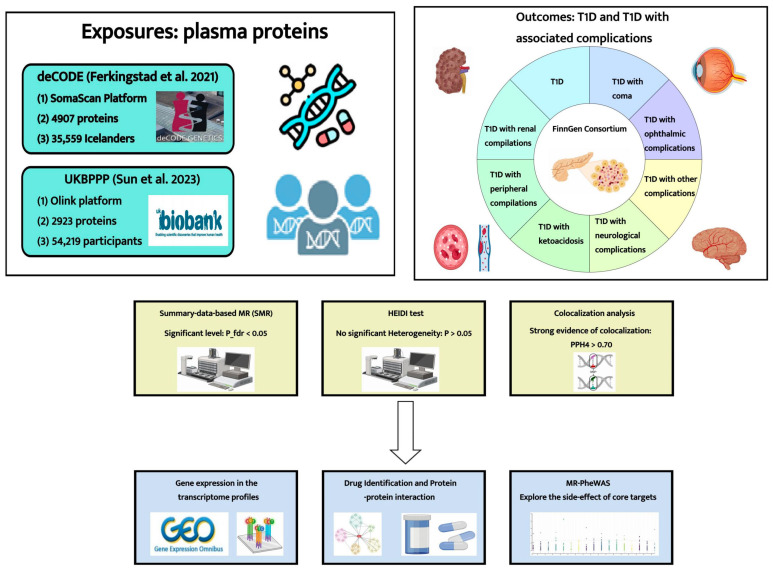
Study design for identification of potential protein biomarkers and therapeutic targets for T1D and its associated complications. UKBPPP, UK Biobank Pharma Proteomics Project; T1D, type 1 diabetes; P_fdr, corrected p-value; HEIDI, Heterogeneity in Dependent Instruments; PPH4, posterior probability of hypothesis 4; MR-PheWAS, MR phenome-wide association study [14,15].

In this study, we leveraged data on plasma proteins from two large-scale studies to unveil potential biomarkers and therapeutic targets for T1D and its associated complications. Through a proteome-wide SMR analysis, we identified candidate plasma proteins. Furthermore, Bayesian colocalization analysis was utilized to uncover shared genetic signals. In addition, we employed a microarray dataset to compare the expression patterns of the identified targets in T1D patients and healthy individuals. Subsequently, a protein–protein interaction (PPI) network was constructed to depict the interactions between the identified protein targets and the established targets of current T1D medications. To ensure the credibility and safety of the identified protein targets, we conducted an MR phenome-wide association study to evaluate the potential adverse effects of the core targets for T1D and its complications. The detailed study design is illustrated in Figure 1.

## 2. Results

### 2.1. Results of SMR Analysis and HEIDI Test

**Figure 2:**
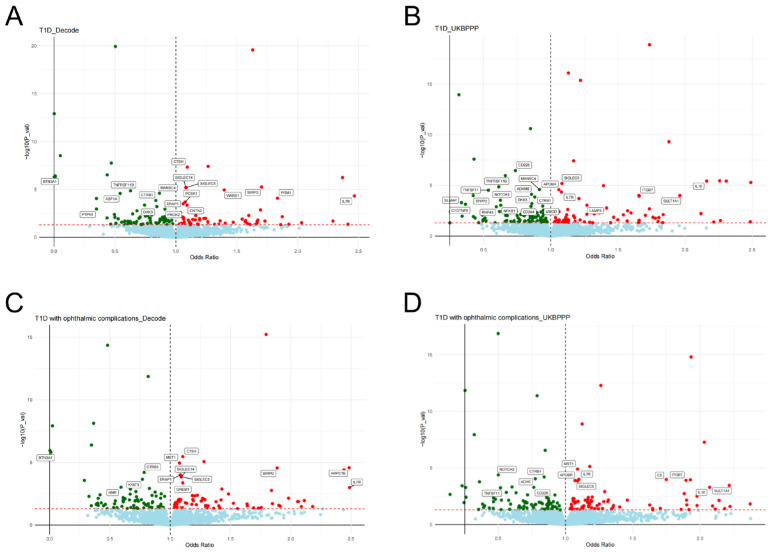
Volcano plots of the SMR results between plasma proteins (crucial targets) and T1D−associated outcomes. (**A**) The exposures were 4907 plasma proteins from the deCODE cohort, and the outcome was T1D; (**B**) the exposures were 2923 plasma proteins from the UKBPPP cohort, and the outcome was T1D; (**C**) the exposures were 4907 plasma proteins from the deCODE cohort, and the outcome was T1D with ophthalmic complications; (**D**) the exposures were 2923 plasma proteins from the UKBPPP cohort, and the outcome was T1D with ophthalmic complications. Red dots represent risk protein targets, green dots represent protective protein targets, and blue dots represent neutral protein targets. Protein targets exhibiting a significant causal association (corrected *p*-value < 0.05 and *p*-value of HEIDI test > 0.01) with a T1D−associated outcome are labeled.

**Figure 3:**
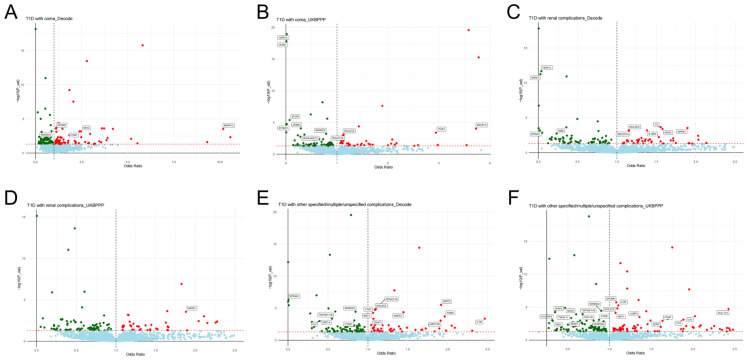
Volcano plots of the SMR results between plasma proteins (crucial targets) and T1D−associated outcomes. (**A**) The exposures were 4907 plasma proteins from the deCODE cohort, and the outcome was T1D with coma; (**B**) the exposures were 2923 plasma proteins from the UKBPPP cohort, and the outcome was T1D with coma; (**C**) the exposures were 4907 plasma proteins from the deCODE cohort, and the outcome was T1D with renal complications; (**D**) the exposures were 2923 plasma proteins from the UKBPPP cohort, and the outcome was T1D with renal complications; (**E**) the exposures were 4907 plasma proteins from the deCODE cohort, and the outcome was T1D with other complications; (**F**) the exposures were 2923 plasma proteins from the UKBPPP cohort, and the outcome was T1D with other complications. Red dots represent risk protein targets, green dots represent protective protein targets, and blue dots represent neutral protein targets. Protein targets exhibiting a significant causal association (corrected *p*-value < 0.05 and *p*-value of HEIDI test > 0.01) with a T1D−associated outcome are labeled.

Significant results (corrected *p*-value < 0.05 and *p*-value of HEIDI test > 0.01) for the causal effects of plasma protein levels on T1D-associated outcomes are presented in Supplementary Tables S3–S17. The volcano plots of the SMR results are illustrated in Figure 2 and Figure 3 and Supplementary Figures S1–S5. After FDR correction, 91 causal associations were identified in the deCODE cohort, and 80 causal associations were identified in the UKBPPP cohort (Supplementary Tables S3–S17). Specifically, we pinpointed 38 potential protein targets for T1D (Supplementary Tables S3 and S11), 15 for T1D with coma (Supplementary Tables S4 and S12), 15 for T1D with ketoacidosis (Supplementary Tables S5 and S13), 5 for T1D with neurological complications (Supplementary Tables S6 and S14), 26 for T1D with ophthalmic complications (Supplementary Tables S7 and S15), 6 for T1D with peripheral circulatory complications (Supplementary Table S8), 13 for T1D with renal complications (Supplementary Tables S9 and S16) and 34 for T1D with other complications (Supplementary Tables S10 and S17). The summarized information about protein targets corresponding to T1D-associated outcomes is presented in Supplementary Table S18.

**Figure 4:**
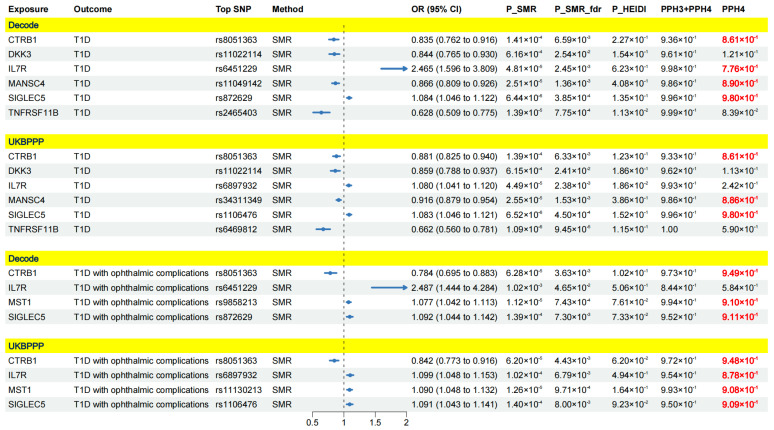
Results of SMR analysis between plasma proteins (crucial targets) and T1D-associated outcomes (T1D and T1D with ophthalmic complications). (Only protein targets demonstrating significant causal associations with outcomes in both the deCODE and UKBPPP cohorts are presented. Red represents significant results of colocalization analysis.)

**Figure 5:**
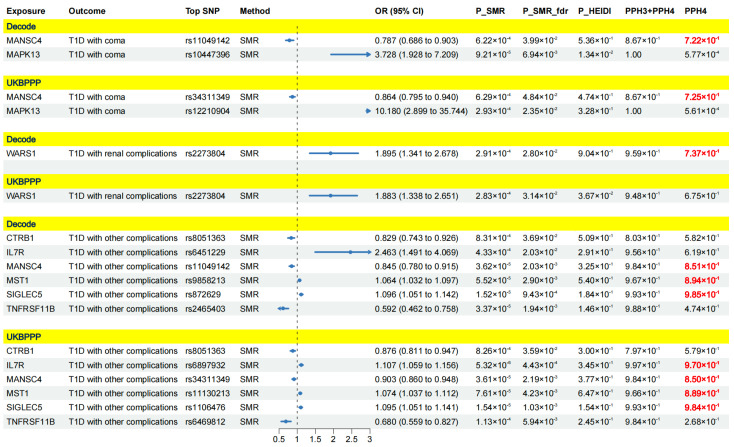
Results of SMR analysis between plasma proteins (crucial targets) and T1D-associated outcomes (T1D with coma, T1D with renal complications and T1D with other complications). (Only protein targets demonstrating significant causal associations with outcomes in both the deCODE and UKBPPP cohorts are presented. Red represents significant results of colocalization analysis.)

After integrating the results, nine proteins were identified as crucial targets for T1D and its complications, as they exhibited causal associations with the outcome across both the deCODE and UKBPPP cohorts. Specifically, there were six crucial protein targets for T1D, four for T1D with ophthalmic complications, two for T1D with coma, one for T1D with renal complications and six for T1D with other complications (Figure 4 and Figure 5).

Specifically, CTRB1, DKK3, IL7R, MANSC4, SIGLEC5 and TNFRSF11B were causally associated with T1D. Among them, CTRB1, DKK3, MANSC4 and TNFRSF11B were associated with a decreased risk of T1D, while IL-7R and SIGLEC5 might increase the risk of T1D. For T1D with ophthalmic compilations, IL7R, MST1 and SIGLEC5 were identified as risk targets, and CTRB1 emerged as a protective target. Additionally, MANSC4 was associated with a decreased risk of T1D with coma, while MAPK13 was associated with an increased risk of T1D with coma. Furthermore, WARS1 might increase the risk of T1D with renal complications. For T1D with other complications, CTRB1, MANSC4 and TNFRSF11B were protective targets, while IL-7R, MST1 and SIGLEC5 were risk targets. The detailed results and forest plots are presented in Figure 4 and Figure 5.

### 2.2. Colocalization Analysis

We conducted a colocalization analysis to ascertain whether the observed causal associations between the nine identified crucial proteins and T1D-associated outcomes were driven by linkage disequilibrium. The results of the colocalization analysis are demonstrated in Figure 4 and Figure 5. PPH4 > 0.7 was considered robust evidence of colocalization. For T1D, four proteins (CTRB1, IL7R, MANSC4 and SIGLEC5) presented strong colocalization in the deCODE cohort, and three proteins (CTRB1, MANSC4 and SIGLEC5) exhibited robust colocalization in the UKBPPP cohort. For T1D with ophthalmic complications, three proteins (CTRB1, MST1 and SIGLEC5) in the deCODE cohort were supported by strong evidence of colocalization. In the UKBPPP cohort, four proteins (CTRB1, IL7R, MST1 and SIGLEC5) had high evidence of colocalization. In the case of T1D with coma, substantial evidence of colocalization was found with MANSC4 in both the deCODE cohort and the UKBPPP cohort. For T1D with renal complications, WARS1 demonstrated robust colocalization in the deCODE cohort. For T1D with other complications, three proteins (MANSC4, MST1 and SIGLEC5) displayed strong colocalization in the deCODE cohort, and four (IL7R, MANSC4, MST1 and SIGLEC5) proteins exhibited strong colocalization in the UKBPPP cohort. The detailed results of the colocalization analysis are presented in Supplementary Table S19. The regional association plots depicting the associations between the identified proteins and outcomes (PPH4 > 0.7) are illustrated in Supplementary Figures S6–S29.

Finally, we identified nine crucial protein targets for T1D and its associated complications. Among them, five were risk targets (IL7R, SIGLEC5, MAPK13, WARS1 and MST1) and four were protective targets (CTRB1, DKK3, MANSC4 and TNFRSF11B). According to the criteria aforementioned, MANSC4, CTRB1, SIGLEC5 and MST1 were classified as tier 1 targets. IL7R and WARS1 were categorized as tier 2 targets. DKK3, TNFRSF11B and MAPK13 were tier 3 targets. The remaining 63 proteins were tier 4 targets.

### 2.3. Expression of Identified Proteins in T1D Patients and Healthy Individuals

**Figure 6:**
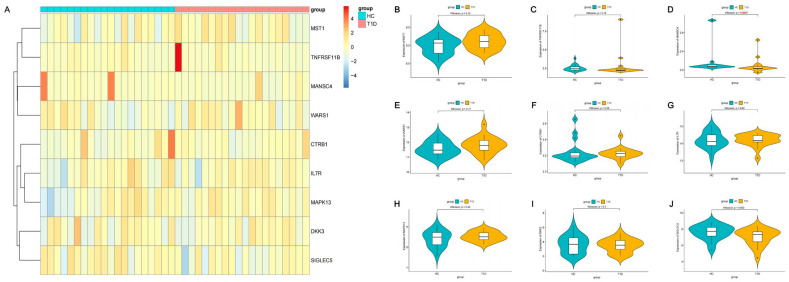
The expression patterns of the corresponding genes of 9 identified crucial protein targets in a microarray dataset. (**A**) The heatmap of the corresponding genes of 9 identified protein targets detected between the T1D and control groups. Red and blue grids represent up- and downregulated genes, respectively. (**B**–**J**) The expression differences in the 9 genes between the two groups.

The expression patterns of the nine identified protein targets were visualized as a heatmap (Figure 6A), with red and blue grids representing upregulated and downregulated genes. Within the heatmap, an ascending trend in the expression levels of risk targets such as MST1, WARS1 and MAPK13 was observed in the T1D group in comparison to the control group. Meanwhile, protective targets like CTRB1, DKK3 and MANSC4 displayed a declining trend in expression. In terms of statistical significance, the expression level of MANSC4 was significantly reduced in the T1D group compared to the control group (Figure 6D, *p* = 0.0047), indicating a potential correlation between MANSC4 and the onset as well as the progression of T1D.

### 2.4. Associations of Identified Protein Targets with Current T1D Medications

**Figure 7:**
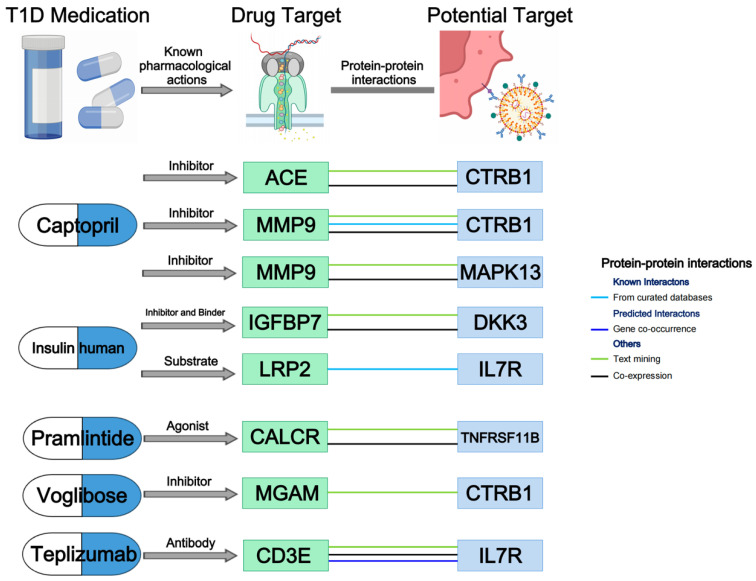
Interactions between current T1D medication targets and identified potential protein targets.

In our endeavor to delineate the current landscape of T1D treatment and explore the interactions among the nine identified protein targets and established therapeutic targets for T1D, we delved into the DrugBank database to obtain the current medications and therapeutic targets for T1D and constructed a PPI network using the STRING database. Finally, we retrieved 11 medications for T1D along with their 20 therapeutic targets, out of which 10 medications had received approval (Supplementary Table S20). The PPI network is presented in Supplementary Figure S30. Figure 7 illustrates the interactions among the identified potential protein targets and established targets. Specifically, CTRB1 was linked to two targets of captopril (ACE and MMP9) and one target of voglibose (MGAM). From the curated database, STRING revealed that IL7R interacted with LRP2 (a therapeutic target of human insulin), which was a reliable interaction. Moreover, IL7R was also associated with CD3E, which is a target of teplizumab. Moreover, MAPK13 was found to be associated with MMP9 (a therapeutic target of captopril), DKK3 with IGFBP7 (a therapeutic target of human insulin) and TNFRSF11B with CALCR (a therapeutic target of pramlintide).

### 2.5. MR Phenome-Wide Association Studies of Core Therapeutic Targets of T1D

**Figure 8:**
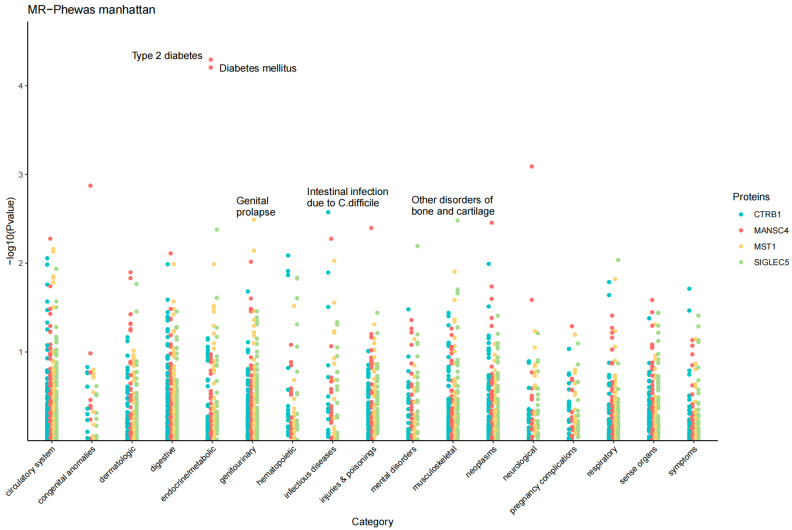
Manhattan plots for MR phenome-wide association study of MANSC4, CTRB1 SILEC5 and MST1. A dot represents a disease or trait.

Given that most medications act on proteins through the blood circulation to exert their effects, we conducted MR phenome-wide association studies to investigate whether the identified protein targets had additional beneficial indications or adverse effects. A total of 783 diseases or traits from the UK Biobank were utilized for the MR screening process. The detailed results are presented in Supplementary Tables S21–S24. The Manhattan plot is demonstrated in Figure 8. Finally, two significant causal associations were identified to be associated with MANSC4. The genetically predicted levels of MANSC4 could reduce the risk of diabetes mellitus (OR = 0.901, 95% CI: 0.856–0.948, *p* = 6.22$\;\times \;10^{-5}$) and type 2 diabetes (OR = 0.898, 95% CI: 0.853–0.946, *p* = 5.07$\;\times \;10^{-5}$). No significant heterogeneity or horizontal pleiotropy was detected in Cochran’s Q test and the MR-Egger test (Supplementary Table S25). For CTRB1, SIGLEC5 and MST1, no additional beneficial indication or adverse effect was detected, which indicated that these protein targets were reliable and safe.

## 3. Discussion

To our knowledge, this is the first study to integrate SMR analysis, Bayesian colocalization analysis, transcriptome analysis and MR phenome-wide association analysis to unveil novel therapeutic protein targets for T1D and its complications, with the hope to provide some preclinical clues for drug development. The SMR analysis identified 171 significant causal associations between the plasma proteins and T1D-associated outcomes, and 72 proteins were regarded as potential targets. By conducting a colocalization analysis to identify the possible effects of linkage disequilibrium, we finally identified nine proteins as crucial targets. Specifically, high genetically predicted levels of five proteins (IL7R, SIGLEC5, MAPK13, WARS1 and MST1) and lower levels of four proteins (CTRB1, DKK3, MANSC4 and TNFRSF11B) were associated with an increased risk of T1D and its associated complications.

In this study, we employed T1D and T1D with different complications as outcomes to identify therapeutic targets for these conditions. Among the 72 identified protein targets, there were 38 targets for T1D, 15 for T1D with coma, 15 for T1D with ketoacidosis, 5 for T1D with neurological complications, 26 for T1D with ophthalmic complications, 6 for T1D with peripheral circulatory complications, 13 for T1D with renal complications and 34 for T1D with other complications. Such a study design allowed us to unearth therapeutic targets for individuals with T1D across diverse clinical scenarios, thus delivering enhanced targeted therapy for T1D patients with various complications. For the nine crucial proteins that exhibited causal associations in both cohorts, the Bayesian colocalization analysis identified shared causal variants and provided evidence for the classification hierarchy. Collectively, four proteins (MANSC4, CTRB1, SIGLEC5 and MST1) were identified as tier 1 targets, two proteins (IL7R and WARS1) were categorized as tier 2 targets and the remaining three proteins (DKK3, TNFRSF11B and MAPK13) were tier 3 targets. Through transcriptome analysis, we further elucidated the different expression patterns of the identified protein targets in T1D patients and healthy individuals. Notably, a marked decrease in the expression of MANSC4, a protective target in T1D patients, was observed, hinting at the potential roles of MANSC4 in the onset as well as the progression of T1D. Through the construction of a PPI network, we unraveled the interactions between CTRB1, MAPK13, DKK3, IL7R and TNFRSF11B and the established therapeutic targets of T1D. Therefore, further understanding the roles of these proteins in the onset and progression of T1D, as well as how existing T1D medications influence protein–protein interactions, will pave the way for novel drug development. MR phenome-wide association studies could delve deeper into the causal associations between the identified protein targets and common diseases, thus identifying their potential beneficial indications and side effects [20]. This methodology could demonstrate the safety and reliability of the targets, thereby maximizing their therapeutic efficacy. Based on the results of the MR analysis, it became apparent that the identified tier 1 targets (MANSC4, CTRB1, SIGLEC5 and MST1) were all secure and reliable. Among them, MANSC4 emerged as a pivotal target that could reduce the risk of diabetes, as the genetically predicted levels of MANSC4 exhibited significant causal associations with a decreased risk of both type 1 and type 2 diabetes. A previous study has already recognized MANSC4 as a therapeutic target for T2D via MR analysis [21], and our research further substantiates and deepens this finding.

MANSC4 is a member of a motif at the N terminus with seven cysteines (MANSC), which is a domain with a well-conserved seven-cysteine motif [22]. In our study, MANSC4 was identified as a tier 1 target for T1D, T1D with coma and T1D with other complications. In Yuan et al.’s study, MANSC4 was pinpointed as a therapeutic target for T2D via MR analysis. Moreover, their investigation revealed connections between MANSC4 and diabetic complications such as diabetic retinopathy, diabetic ketoacidosis and diabetic hypoglycemia [21]. Nevertheless, their study did not specify whether the complications associated with MANSC4 were related to T1D or T2D, thus posing certain limitations. Our research further validated and enhanced this finding, establishing the causal associations between MANSC4 and T1D as well as its complications through SMR analysis and colocalization analysis. Currently, research on MANSC4 remains somewhat limited. Lafleur et al. highlighted the potential link between MANSC4 and inflammation responses triggered by periodontitis within a hyperglycemic microenvironment [23]. As we know, periodontitis is a prevalent complication of diabetes. Based on the existing findings, we speculate that MANSC4 has the potential to serve as a pivotal node in unraveling the relationship between T1D and periodontitis and become a crucial therapeutic target.

Chymotrypsinogen B1 (CTRB1) is a member of the serine protease family of enzymes and forms a principal precursor of the pancreatic proteolytic enzymes [24]. Therefore, CTRB1 may be closely related to pancreatic diseases. In our study, CTRB1 was identified as a crucial protective target for T1D, T1D with ophthalmic complications and T1D with other complications. Furthermore, we have also observed connections between CTRB1 and the risk of other pancreatic disorders, such as alcoholic and non-alcoholic pancreatitis, in previous studies [24,25]. Inshaw et al. reported that CTRB1 served as a shared genetic link between T1D and T2D, but it exerted opposite influences on the risks of the two forms of diabetes [26]. Hart et al.’s study demonstrated that the CTRB1/2 genetic locus was associated with a reduced response to treatment with dipeptidyl peptidase 4 inhibitors in T2D patients [27]. These findings provide further validation of our finding that CTRB1 might mitigate reduce the risk of T1D and its complications.

Sialic acid binding Ig-like lectin 5 (SIGLEC5) is a member of the sialic acid-binding immunoglobulin-like lectin (Siglec) family, known for its pivotal roles in modulating the immune response [28]. In our study, SIGLEC5 emerged as a crucial protein target for T1D, T1D with ophthalmic complications and T1D with other complications. In addition, SIGLEC5 was also associated with T1D with coma, as well as T1D with renal complications. Li et al. highlighted SIGLEC5 as a novel biomarker for diabetic patients with critical limb ischemia (CLI), potentially serving as an early diagnostic indicator. At present, investigations into SIGLEC5 and T1D remain relatively limited, emphasizing the significant untapped potential for the development of targeted medications. However, a number of studies have highlighted a substantial association between SIGLEC5 and the risk of periodontitis [29,30,31,32]. Consequently, similarly to MANSC4, SIGLEC5 might offer novel insights into the intricate interplay between T1D and periodontitis.

Mammalian sterile 20-like kinase 1 (MST1), also known as serine/threonine protein kinase 4 (STK4), is a member of the Ste-20 family and is primarily distributed in the cytoplasm [33]. In our study, genetically predicted levels of MST1 were causally associated with an elevated risk of T1D with ophthalmic complications and T1D with other complications, exhibiting robust colocalization. Currently, MST1 exhibits therapeutic potential across various fields, holding promise as a therapeutic target for conditions such as diabetes, cardiac diseases, ischemia–reperfusion injury and other conditions [34,35,36]. It is widely recognized that the dysfunction of islet β-cells constitutes a crucial factor in the onset of T1D [37], and previous studies have demonstrated that the overexpression of MST1 can detrimentally impact islet β-cells, inducing their apoptosis [34]. Ardestani et al. demonstrated that MST1 amplified the activation of caspases, thereby upregulating Bcl-2-like protein 11 (BIM) and promoting the apoptosis of islet β-cells [38]. Moreover, MST1 could directly phosphorylate pancreatic and duodenal homeobox 1 (PDX1), a crucial β-cell transcription factor, thus ubiquitinating and degrading PDX1. This process ultimately contributes to the dysfunction of islet β-cells [38,39,40]. Our study further elucidated the causal relationship between MST1 and T1D as well as its complications. Regrettably, no approved pharmaceutical interventions targeting this protein were retrieved from the DrugBank database. Looking ahead, drug development targeting MST1 holds promise in offering renewed optimism to T1D patients.

This study has several strengths. Firstly, we conducted proteome-wide SMR and colocalization analysis to identify potential causal protein targets for T1D and its complications. The MR design effectively mitigates the interference of confounding factors and reverse causal bias, thereby establishing definitive causal relationships [41]. Colocalization analysis could help to eliminate the potential bias caused by linkage disequilibrium, fortifying the robustness of our findings [42]. Secondly, instead of using conventional two-sample MR analysis, we conducted SMR analysis, which could reach much higher statistical power [16]. Hence, employing SMR analysis would be more conducive to therapeutic target exploration. Thirdly, we utilized the latest two large-scale proteomic datasets (from deCODE genetics and the UK Biobank) for analysis and mutual validation, thereby further bolstering the reliability of the results. Furthermore, at present, there are few MR studies focusing on the complications of T1D. Our research delved into T1D and its common complications, identifying precise therapeutic targets for a spectrum of outcomes, which holds promise in advancing clinical management and drug development for T1D. Lastly, the transcriptome analysis and PPI network offered valuable insights into the potential pathogenic effects of the identified targets, and the MR phenome-wide association study further validated their safety.

However, it is crucial to acknowledge certain limitations of our study. Firstly, all of the data utilized in this study were derived from European individuals. While this choice minimized the population stratification bias, the generalization of our findings to other racial or ethnic groups warrants further exploration and discussion. Secondly, the sample size of GWAS on T1D and its complications is still limited, thereby potentially compromising the statistical power. In the future, once larger-scale GWAS data are available, we will promptly update our research findings. In addition, we only evaluated the role of genetically predicted levels of plasma proteins in T1D, whereas the association between the protein levels in islet cells and T1D potentially holds greater significance for clinical guidance. Regrettably, the absence of data impeded our efforts to compensate for this deficiency. Finally, we did not conduct cellular or animal experiments to investigate the specific mechanisms driving these identified protein targets to increase or decrease the risk of T1D and its complications. Subsequent investigations can build on our research findings to explore specific molecular mechanisms and pave the way for targeted therapy for T1D.

## 4. Materials and Methods

### 4.1. Data Sources

We obtained summary statistics on plasma protein levels from two extensive proteome-wide studies [14,15]. Ferkingstad et al. provided pQTL data on 4907 plasma proteins from 35,559 Icelanders (deCODE genetics) [14], and Sun et al. provided pQTL data for 2923 plasma proteins from 54,219 participants (UK Biobank Pharma Proteomics Project (UKBPPP)) [15].

The GWAS summary statistics on T1D and T1D with complications were extracted from the R9 release results from the FinnGen consortium. The data encompassed T1D (8967 cases and 308,373 controls), T1D with coma (2050 cases and 308,280 controls), T1D with ketoacidosis (2102 cases and 308,280 controls), T1D with neurological complications (1077 cases and 308,280 controls), T1D with ophthalmic complications (5202 cases and 308,280 controls), T1D with peripheral circulatory complications (669 cases and 308,280 controls), T1D with renal complications (1579 cases and 308,280 controls) and T1D with other complications (6234 cases and 308,280 controls).

It is important to note that the exposure data originated from Iceland and the UK, while the outcome data were sourced from Finland. Consequently, there was no sample overlap between the exposure and outcome. All information on the summary statistics datasets utilized in this study is listed in Supplementary Table S1.

### 4.2. Summary-Data-Based MR (SMR) Analysis

We conducted summary-data-based Mendelian randomization (SMR) analysis to evaluate the causal associations between the plasma protein levels and outcomes. Firstly, common (minor allele frequency (MAF) > 0.01) pQTL single-nucleotide polymorphisms (SNPs) significantly (*p* < 5$\;\times \;10^{-8}$) associated with plasma protein levels were selected. Subsequently, we selected the top associated cis-pQTLs (within a ±1000 kb window centered around the corresponding genes) of proteins as IVs. In addition, SNPs with allele frequency differences larger than 0.2 between any pairwise datasets (LD reference sample, pQTL summary data and outcome summary data) were excluded [17]. The Heterogeneity in Dependent Instruments (HEIDI) test was also applied to evaluate whether the observed causal association was caused by a linkage scenario. A *p*-value in the HEIDI test < 0.01 indicated the existence of linkage disequilibrium and pleiotropy. Both the SMR analysis and HEIDI test were conducted using the SMR software (V.1.3.1) [16]. The statistical results of the SMR analysis were demonstrated in the form of odds ratios (OR) and 95% confidence intervals (95% CI), with a nominal significance threshold of a *p*-value < 0.05. To avoid false positive results, we adjusted the *p*-values to control the false discovery rate (FDR) at α = 0.05 [43]. Proteins with *p*-values corrected by the FDR method < 0.05 and *p*-values in the HEIDI test > 0.01 were regarded as causal proteins of the outcome.

### 4.3. Bayesian Colocalization Analysis

We conducted colocalization analysis using the R package “coloc” (V.5.2.3) to confirm that the 9 identified crucial proteins and T1D-associated outcomes might share the same causal variant [44]. The prior probabilities that the SNP was associated with only trait 1, only trait 2 and both traits were set at $P_{1}$ = 1$\;\times \;10^{-4}$, $P_{2}$ = 1$\;\times \;10^{-4}$, $P_{3}$ = 1$\;\times \;10^{-5}$, respectively. Moreover, there were five hypotheses in the Bayesian colocalization analysis: (1) H0—there is no causal variant with the two traits; (2) H1—there is a causal variant associated with exclusively trait 1; (3) H2—there is a causal variant associated with only trait 2; (4) H3—both traits have a causal variant, but they are distinct; (5) H4—both traits have a causal variant, and they share the same one. Each hypothesis had a posterior probability (PP). The identified proteins and the outcome were considered to have substantial evidence of colocalization if the posterior probability of hypothesis 4 (PPH4) was > 0.7 [17]. In addition, PPH3 + PPH4 > 0.7 was regarded as median evidence of colocalization (both the exposure and outcome had a causal SNP) [45]. The regional association plots were visualized using the package “LocusCompareR” (V.1.0.0) [46].

### 4.4. Integrating Results from Promote-Wide SMR Analysis (Classification Hierarchy)

Proteins exhibiting a significant causal association (corrected *p*-value < 0.05 and *p*-value of HEIDI test > 0.01) with T1D-associated outcomes were identified as potential targets. If the causal relationship between the protein and the outcome was verified in both the deCODE and UKBPPP cohorts, it was regarded as a crucial protein target. We further categorized the 9 identified crucial protein targets for T1D based on the following criteria: (1) the protein (from both the deCODE cohort and the UKBPPP cohort) demonstrated a causal association with the outcome, and the directions of the effects in the two cohorts were consistent (both ORs were greater than 1 or less than 1); (2) the protein (from either the deCODE cohort or the UKBPPP cohort) exhibited strong colocalization with the outcome (PPH4 > 0.7); (3) the protein (from both the deCODE cohort and the UKBPPP cohort) exhibited strong colocalization with the outcome (PPH4 > 0.7). Crucial proteins that satisfied criteria (1) and (3) were classified as tier 1 targets; crucial proteins fulfilling criteria (1) and (2) were categorized as tier 2 targets; crucial proteins meeting only criterion (1) were defined as tier 3 targets; proteins beyond the 9 crucial proteins were considered as tier 4 targets.

### 4.5. Analysis of Expression Patterns of Identified Proteins in T1D Patients and Healthy Individuals

We retrieved a microarray dataset (GSE193273) that represented the transcriptome profiles of the peripheral blood of 20 T1D patients and 20 healthy individuals from the Gene Expression Omnibus (GEO) database (https://www.ncbi.nlm.nih.gov/geo/, accessed on 13 March 2024) [47]. We used the Wilcoxon test to investigate whether significant differences existed in the expression of genes corresponding to the identified protein targets between T1D patients and healthy individuals. The R package “pheatmap” was utilized to visualize the expression patterns of the genes corresponding to the identified 9 crucial protein targets in the T1D group and control group. The R package “ggpubr” was used to visualize the expression differences of the 9 genes between the two groups.

### 4.6. Drug Identification and Protein–Protein Interaction (PPI) Network

In order to further advance the clinical translation of the identified protein targets, we searched for established therapeutic targets of T1D and constructed a protein–protein interaction (PPI) network. Firstly, we delved into the DrugBank database (V.5.0, https://go.drugbank.com, accessed on 17 March 2024) to explore existing drugs along with their targets for the treatment of T1D [48]. The Search Tool for the Retrieval of Interacting Genes (STRING, V.12.0, https://string-db.org/, accessed on 20 March 2024) database was used to construct a PPI network among the 9 identified protein targets and existing T1D drug targets, with the minimum required interaction score set at 0.4 (median confidence) [49].

### 4.7. MR Phenome-Wide Association Study

To further delve into the potential beneficial indications or adverse effects of the core protein targets (4 tier 1 targets: MANSC4, CTRB1, SIGLEC5 and MST1), we conducted MR phenome-wide association studies. The cis-pQTLs of the four protein targets in the deCODE cohort were employed as exposures, which satisfied the following criteria: (1) the pQTLs exhibited a genome-wide significant association (*p* < 5$\;\times \;10^{-8}$); (2) SNPs should be independent to avoid linkage disequilibrium ($r^{2}$ = 0.001); (3) the pQTLs should be cis-pQTLs (within a ±1000 kb window centered around the corresponding genes); (4) the F-statistics of IVs must be greater than 10 to avoid weak instrument bias [50]. The summary statistics of disease or traits from the UK Biobank cohort (sample size up to 408,961) served as outcomes. These GWASs from the UK Biobank were processed using the Scalable and Accurate Implementation of Generalized Mixed Model (SAIGE V.0.29) method, and they were adjusted for imbalanced case–control ratios [51]. All 1403 diseases or traits were defined based on the International Classification of Diseases and Related Health Problems (ICD-9/-10) [41]. To ensure sufficient statistical power, 783 traits (diseases) with more than 500 cases were selected from the 1403 traits (diseases) as outcomes (Supplementary Table S2). In the MR analysis, when there were two or more IVs available, the inverse-variance weighted (IVW) method was used [52]. If there was only 1 SNP available, the Wald ratio (WR) method was employed [53]. To avoid false positive results, we adjusted the *p*-values to control the false discovery rate (FDR) at α = 0.05 [43]. We also employed Cochran’s Q test and the MR-Egger intercept test to assess the heterogeneity and horizontal pleiotropy (*p*-value < 0.05 indicated statistically significant heterogeneity or horizontal pleiotropy) [54,55]. The additional causal effects were considered when the corrected *p*-value of the IVW or WR method was < 0.05 without significant heterogeneity or horizontal pleiotropy.

### 4.8. Data Analysis

All of the analyses were conducted in the SMR software (V.1.3.1) or R software (V.4.3.1), with *p* < 0.05 considered statistically significant.

## 5. Conclusions

In summary, this proteome-wide SMR analysis identifies many protein targets causally associated with T1D and its complications. Additionally, the colocalization analysis enhances the robustness of our findings and helps to classify these proteins into four tiers. The top-tier (tier 1) targets (MANSC4, CTRB1, SIGLEC5 and MST1) are the most promising candidates for drug development. The transcriptome analysis, PPI network and MR phenome-wide association study provide new insights into the mechanisms and safety of crucial protein targets. However, further basic and clinical studies are needed to assess the efficacy of these identified targets and advance the targeted treatment of T1D.

## Data Availability

All data generated or analyzed during this study are included in this published article and its Supplementary Materials. Additional materials can be obtained from the corresponding author upon reasonable request.

## Supplementary Materials

The following supporting information can be downloaded at: https://www.mdpi.com/article/10.3390/ph17060766/s1, Table S1: The sources for all statistical summary datasets used in this study; Table S2: The information of 783 diseases incorporated in the MR-PheWAS analysis; Tables S3–S10: Significant SMR results between plasma proteins (In the deCODE cohort) and T1D-associated outcomes; Tables S11–S18: Significant SMR results between plasma proteins (In the UKBPPP cohort) and T1D-associated outcomes; Table S19: Colocalization results of pQTLs for proteins with GWAS-associated SNPs; Table S20: Current medications and corresponding targets for T1D; Tables S21–S25: MR-PheWAS results; Figures S1–S5: Volcano plots of the SMR results between plasma proteins and T1D-associated outcomes; Figures S6–S29: Regional association plots of colocalization analysis between proteins and T1D-associated outcomes; Figure S30: Protein–protein interaction (PPI) network among identified crucial protein targets and established targets of T1D.
