# Supplementary material for: Novel Protein Biomarkers and Therapeutic Targets for Type 1 Diabetes and Its Complications: Insights from Summary-Data-Based Mendelian Randomization and Colocalization Analysis
Source: Pharmaceuticals (Basel). 2024 Jun 11;17(6):766. doi: 10.3390/ph17060766 (PMC11206317; doi:10.3390/ph17060766)
Supplement: Supplementary file 1 [file pharmaceuticals-17-00766-s001.zip › Figure S1~S30.pdf]

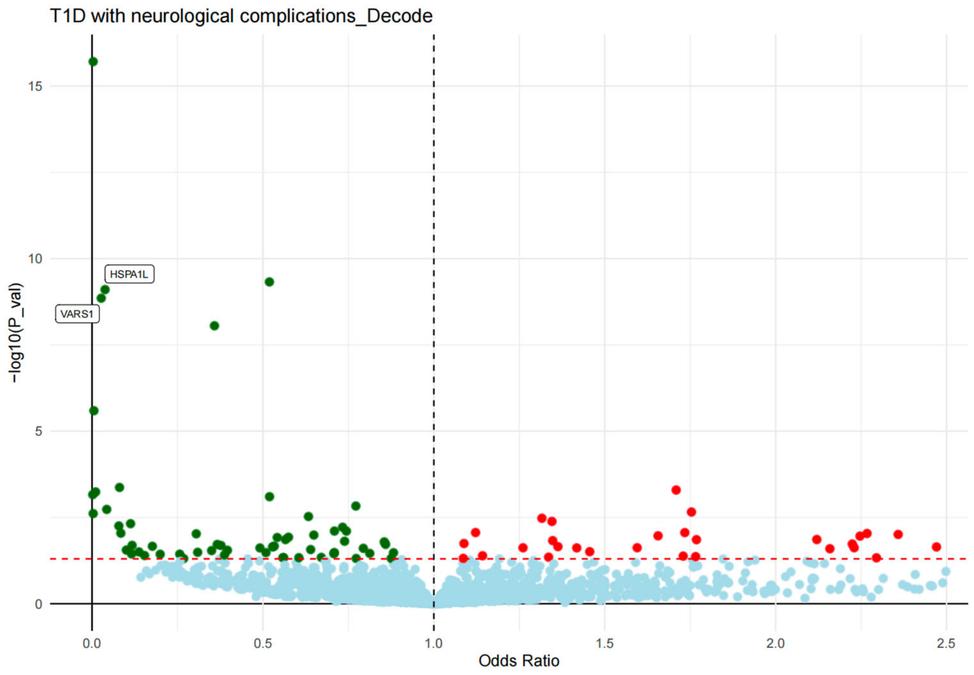

Figure S1 Volcano plots of the SMR results between plasma proteins (In the deCODE cohort) and T1D with neurological complications. Red dots represent risk protein targets, green dots represent protective protein targets, and blue dots represent Neutral Protein targets. Protein targets exhibiting significant causal association (corrected *p*-value < 0.05 and *p*-value of HEIDI test > 0.01) with T1D associated outcome were labeled.

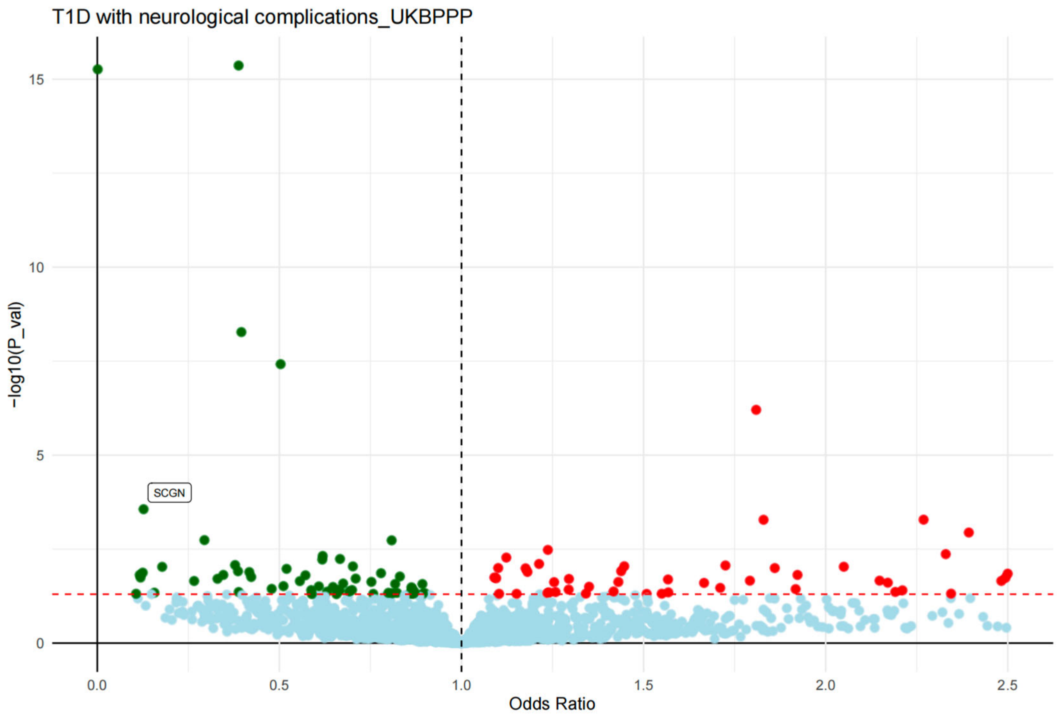

Figure S2 Volcano plots of the SMR results between plasma proteins (In the UKBPPP cohort) and T1D with neurological complications. Red dots represent risk protein targets, green dots represent protective protein targets, and blue dots represent Neutral Protein targets. Protein targets exhibiting significant causal association (corrected *p*-value < 0.05 and *p*-value of HEIDI test > 0.01) with T1D associated outcome were labeled.

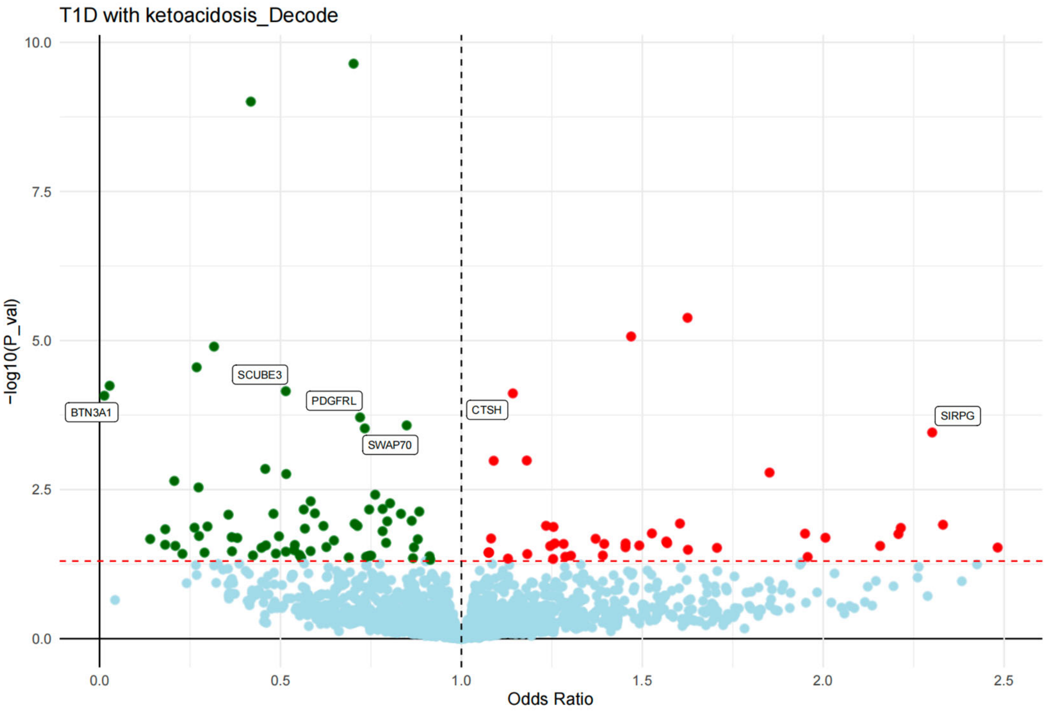

Figure S3 Volcano plots of the SMR results between plasma proteins (In the deCODE cohort) and T1D with ketoacidosis. Red dots represent risk protein targets, green dots represent protective protein targets, and blue dots represent Neutral Protein targets. Protein targets exhibiting significant causal association (corrected *p*-value < 0.05 and *p*-value of HEIDI test > 0.01) with T1D associated outcome were labeled.

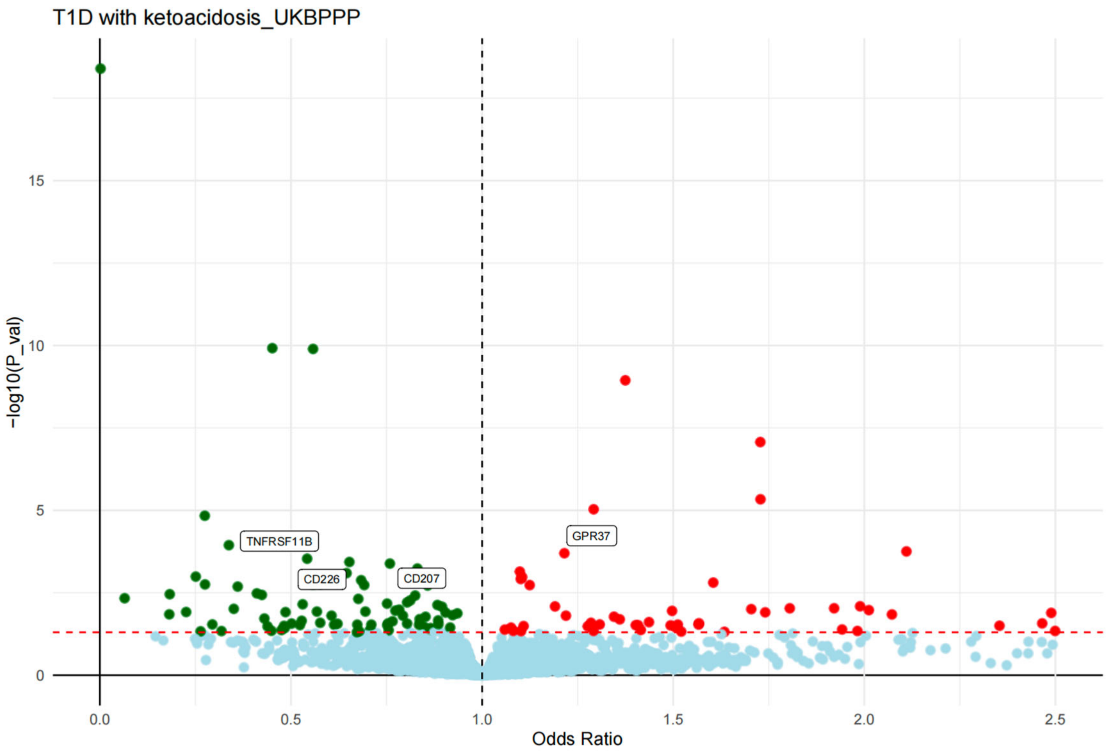

Figure S4 Volcano plots of the SMR results between plasma proteins (In the UKBPPP cohort) and T1D with ketoacidosis. Red dots represent risk protein targets, green dots represent protective protein targets, and blue dots represent Neutral Protein targets. Protein targets exhibiting significant causal association (corrected *p*-value < 0.05 and *p*-value of HEIDI test > 0.01) with T1D associated outcome were labeled.

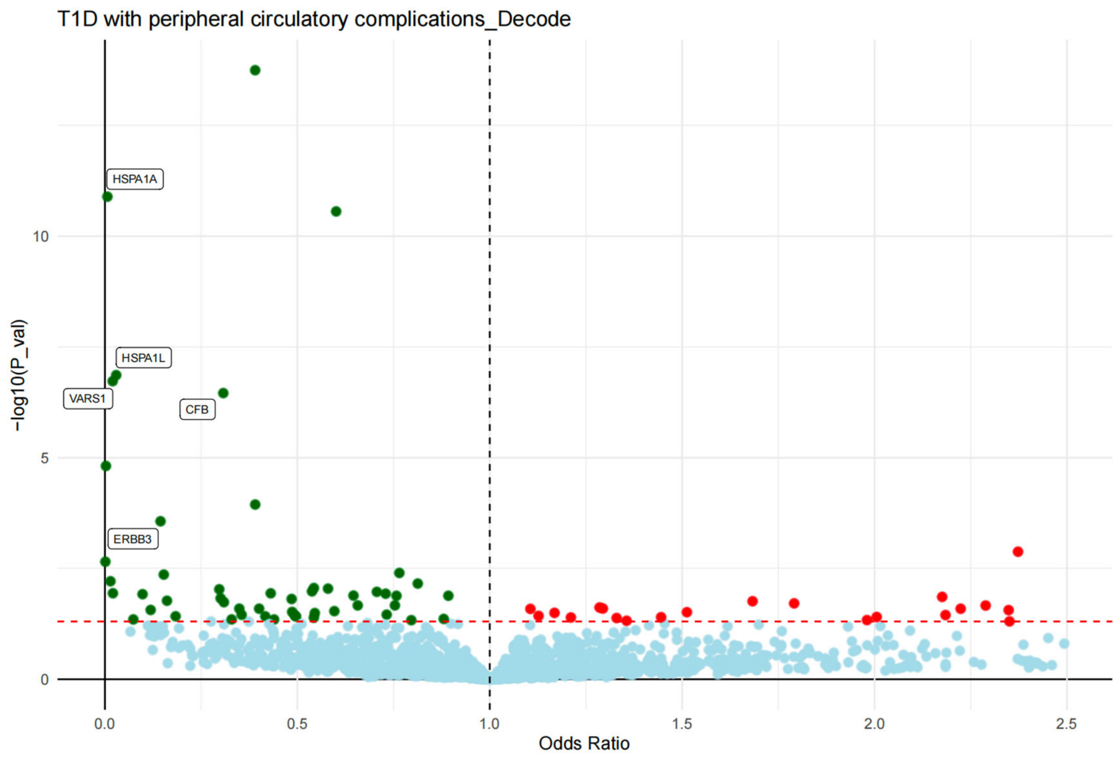

Figure S5 Volcano plots of the SMR results between plasma proteins (In the UKBPPP cohort) and T1D with circulatory complications. Red dots represent risk protein targets, green dots represent protective protein targets, and blue dots represent Neutral Protein targets. Protein targets exhibiting significant causal association (corrected *p*-value < 0.05 and *p*-value of HEIDI test > 0.01) with T1D associated outcome were labeled.

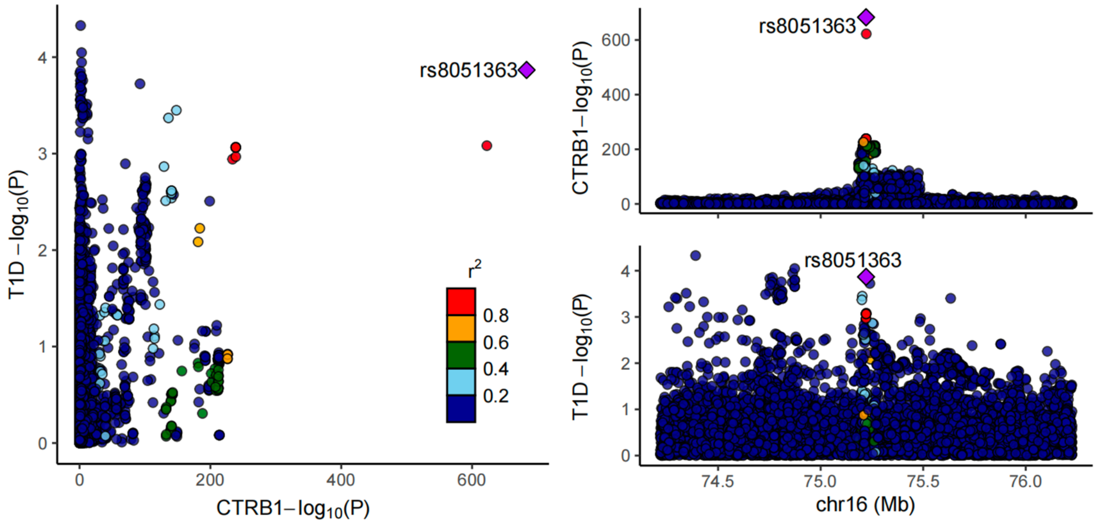

Figure S6 Regional association plots of colocalization analysis between CTRB1 (deCODE) and T1D.

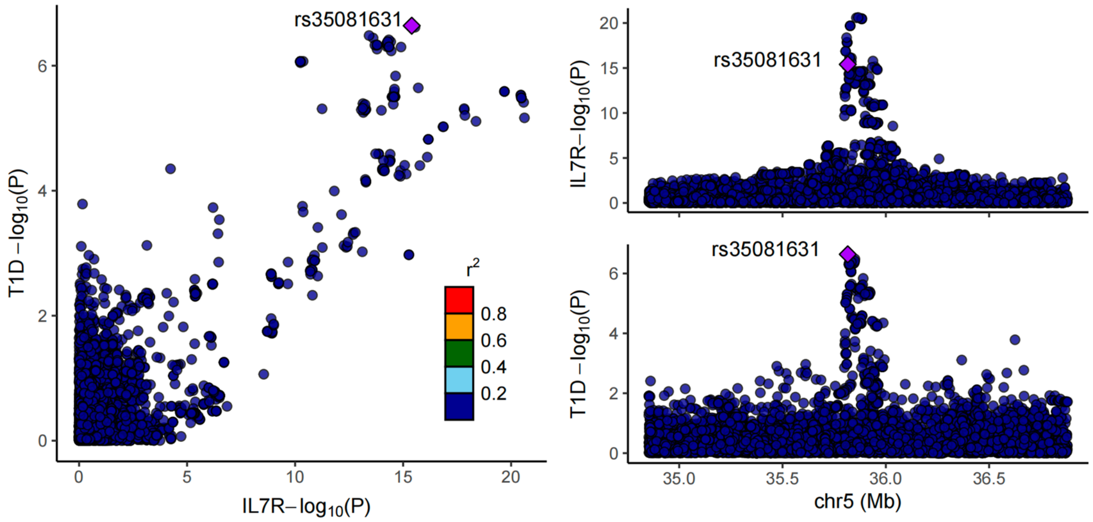

Figure S7 Regional association plots of colocalization analysis between IL7R (deCODE) and T1D.

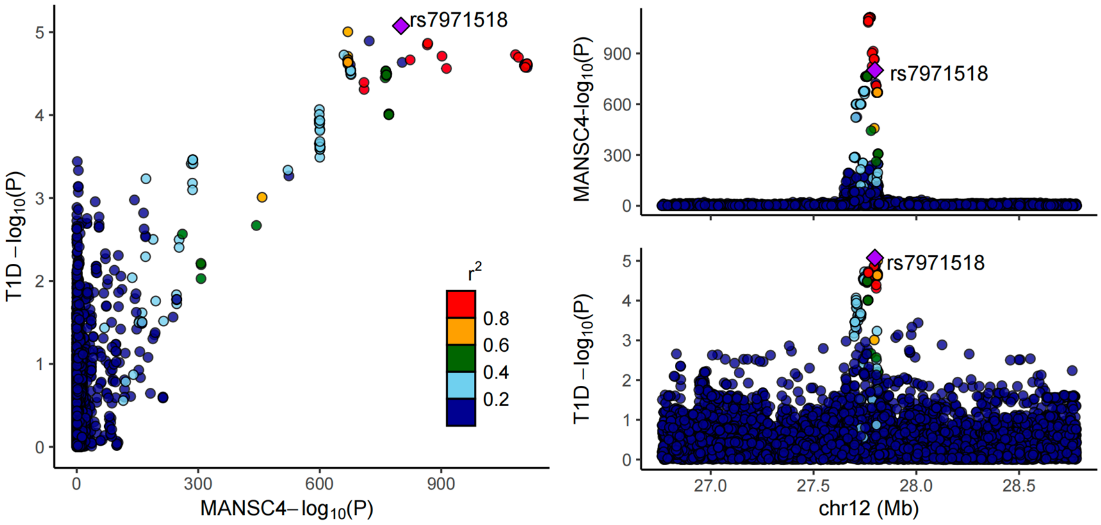

Figure S8 Regional association plots of colocalization analysis between MANSC4 (deCODE) and T1D.

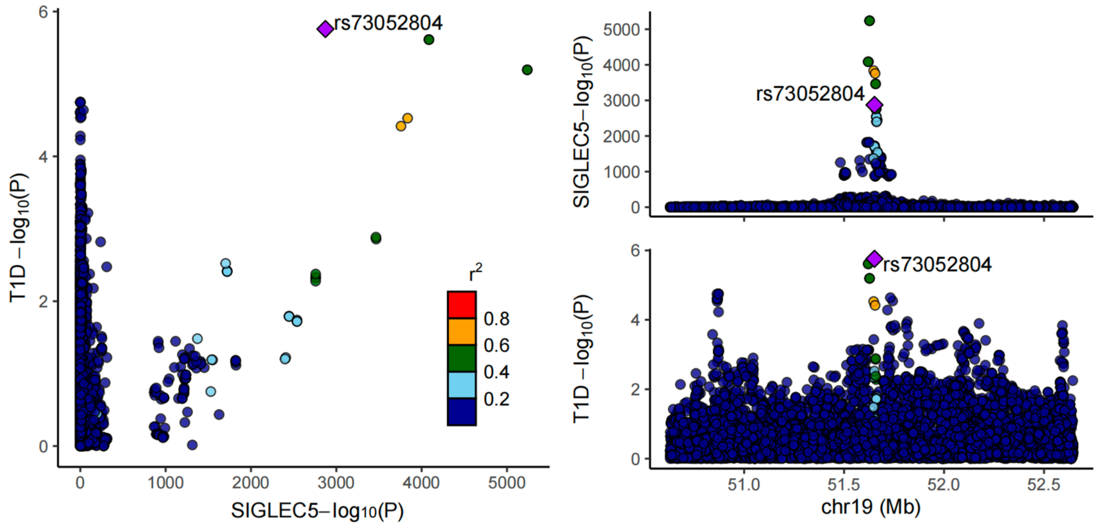

Figure S9 Regional association plots of colocalization analysis between SIGLEC5 (deCODE) and T1D.

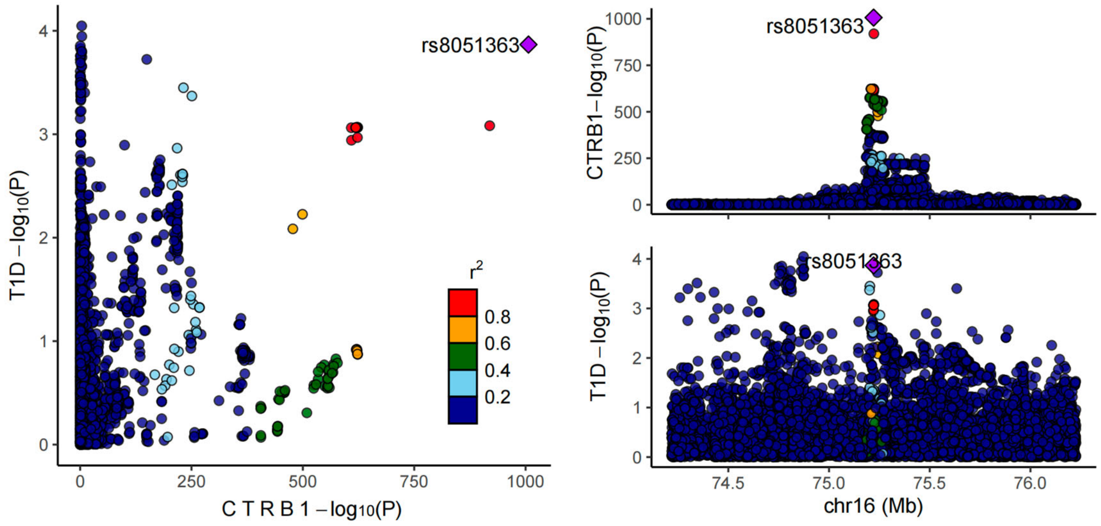

Figure S10 Regional association plots of colocalization analysis between CTRB1 (UKBPPP) and T1D.

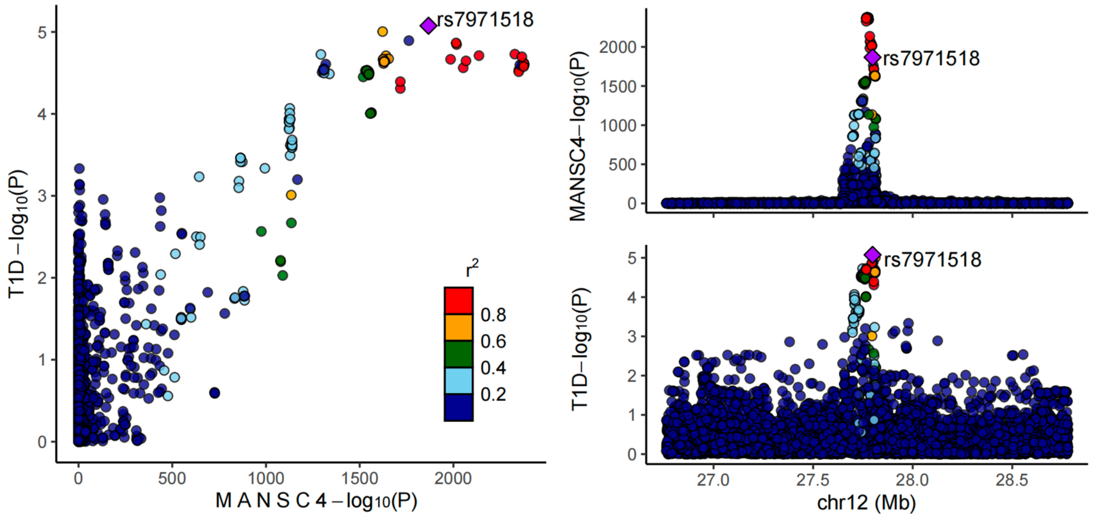

Figure S11 Regional association plots of colocalization analysis between MANSC4 (UKBPPP) and T1D.

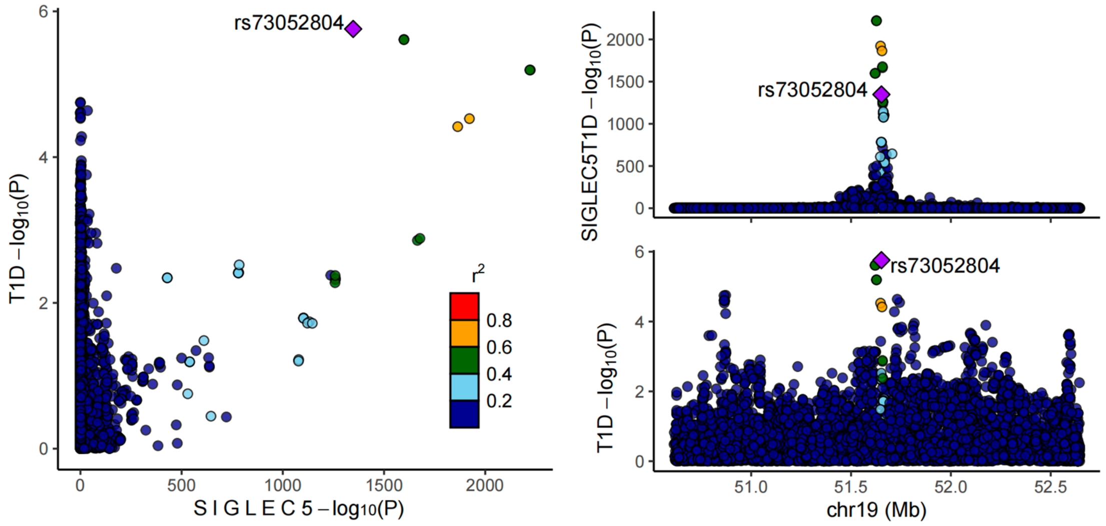

Figure S12 Regional association plots of colocalization analysis between SIGLEC5 (UKBPPP) and T1D.

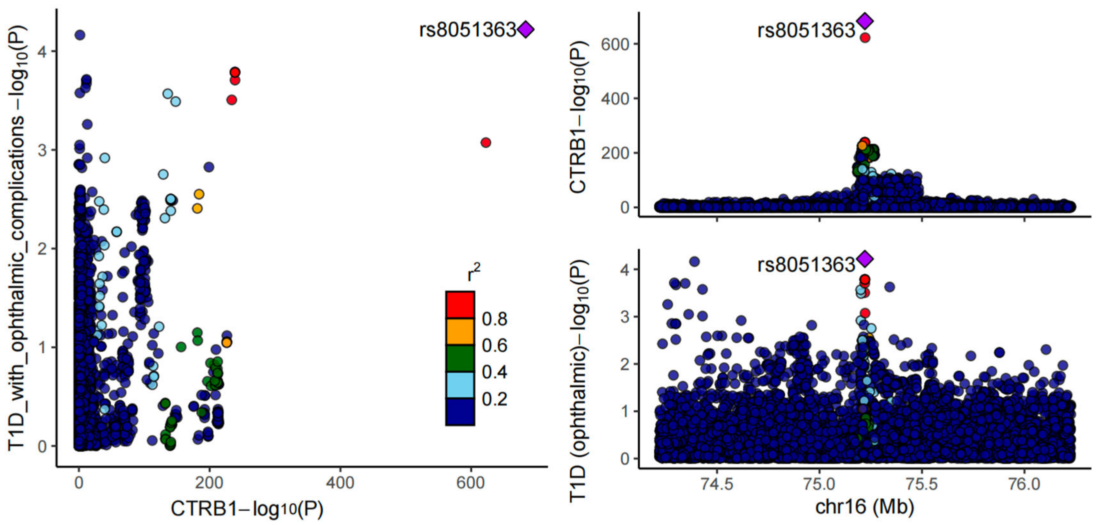

Figure S13 Regional association plots of colocalization analysis between CTRB1 (deCODE) and T1D with ophthalmic complications.

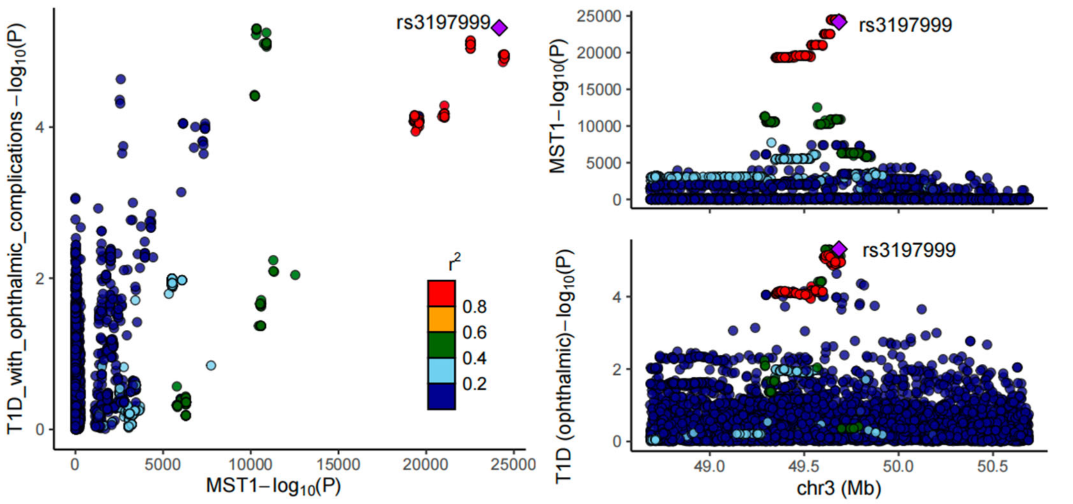

Figure S14 Regional association plots of colocalization analysis between MST1 (deCODE) and T1D with ophthalmic complications.

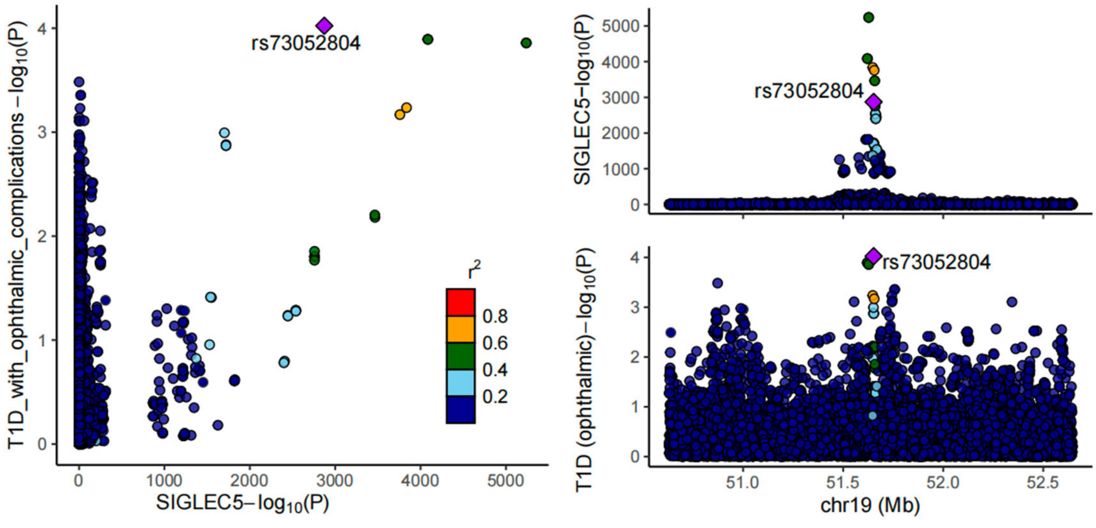

Figure S15 Regional association plots of colocalization analysis between SIGLEC5 (deCODE) and T1D with ophthalmic complications.

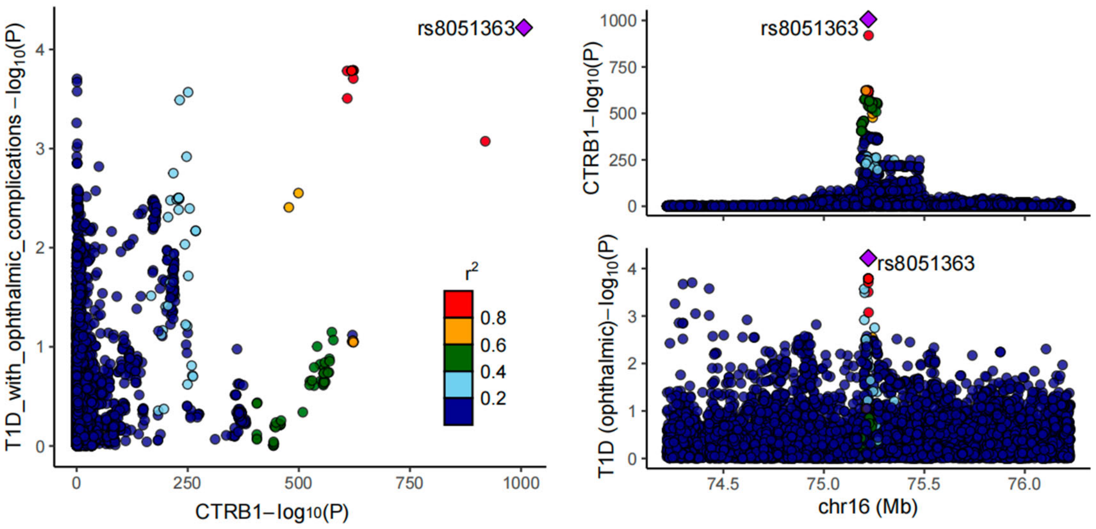

Figure S16 Regional association plots of colocalization analysis between CTRB1 (UKBPPP) and T1D with ophthalmic complications.

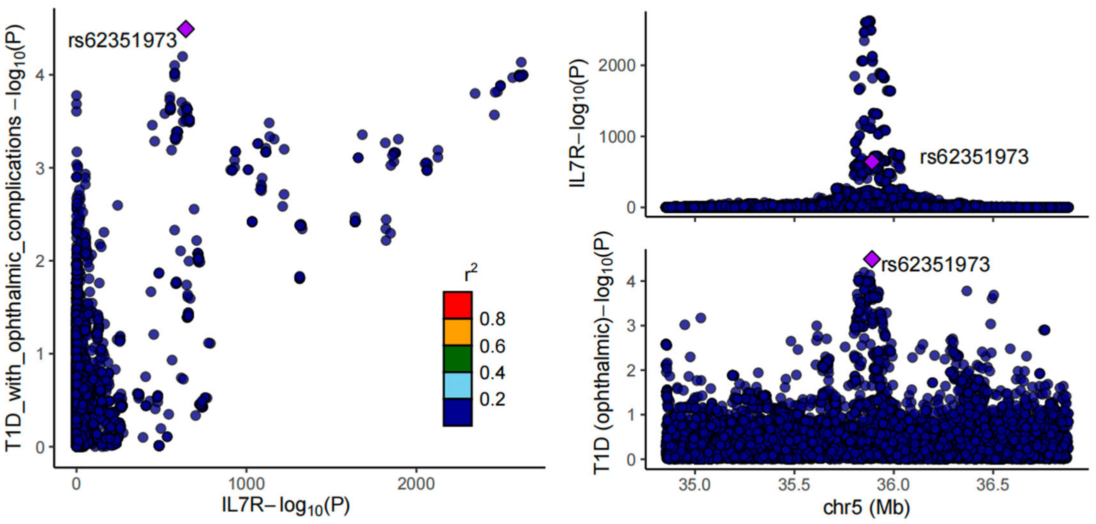

Figure S17 Regional association plots of colocalization analysis between IL7R (UKBPPP) and T1D with ophthalmic complications.

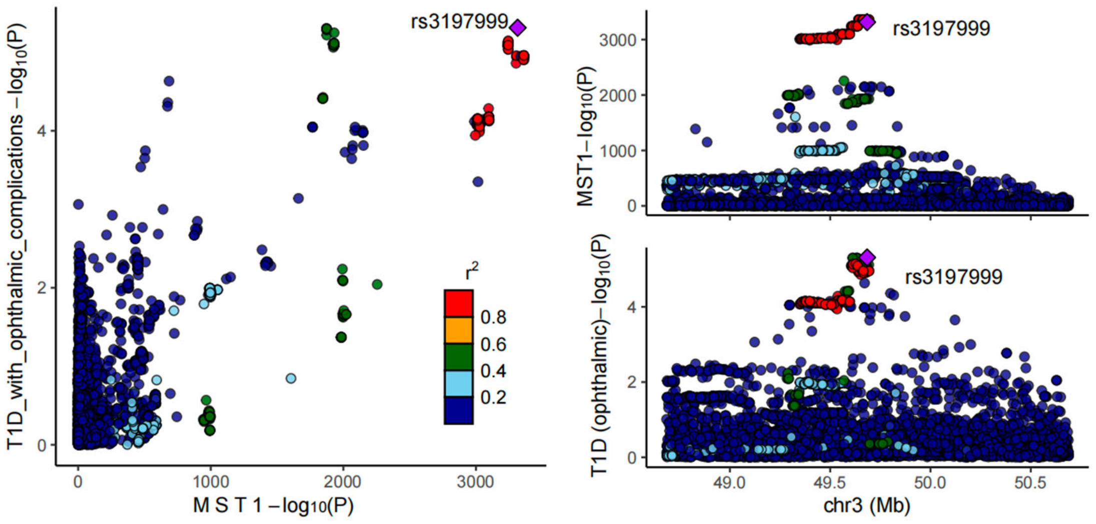

Figure S18 Regional association plots of colocalization analysis between MST1 (UKBPPP) and T1D with ophthalmic complications.

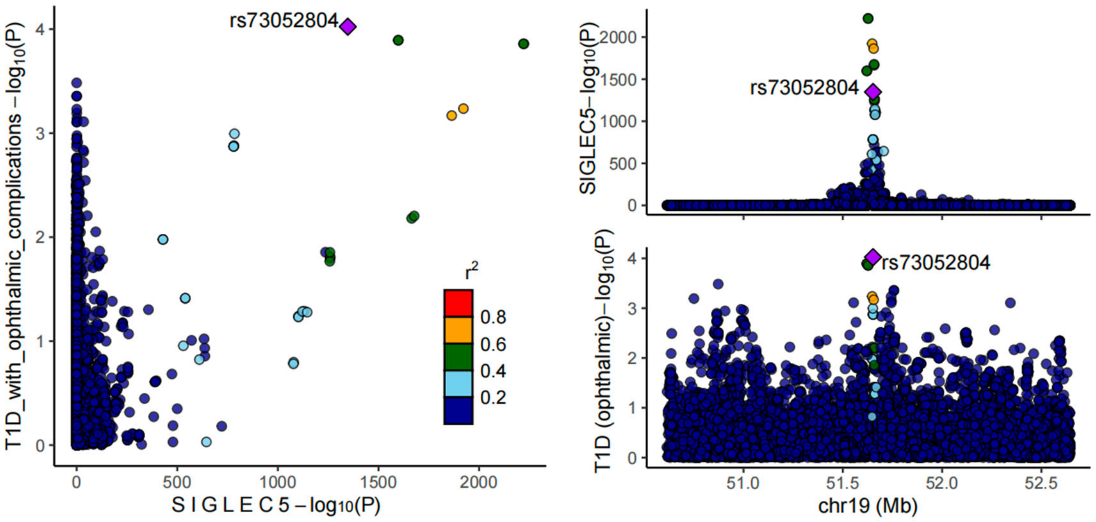

Figure S19 Regional association plots of colocalization analysis between SIGLEC5 (UKBPPP) and T1D with ophthalmic complications.

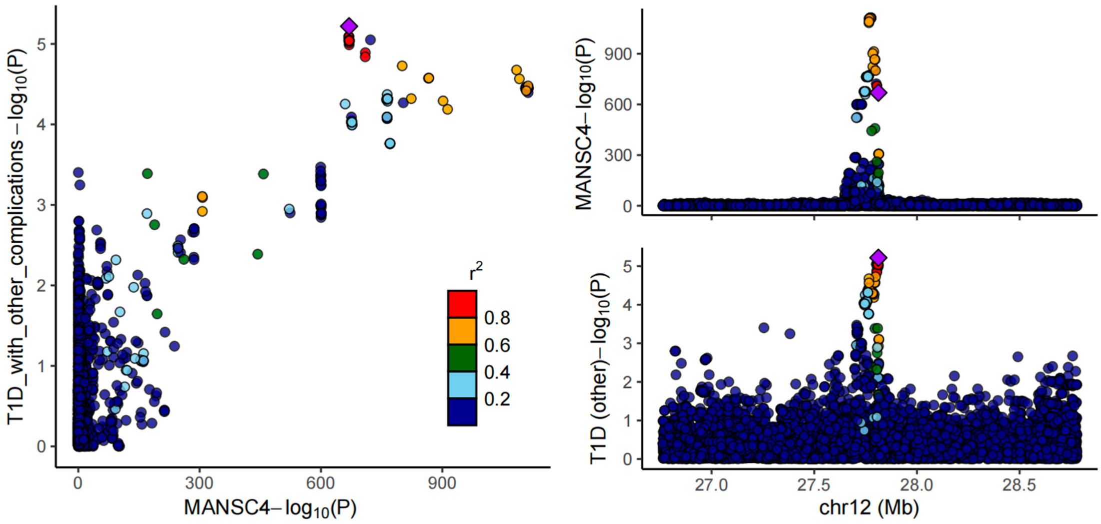

Figure S20 Regional association plots of colocalization analysis between MANSC4 (deCODE) and T1D with other complications.

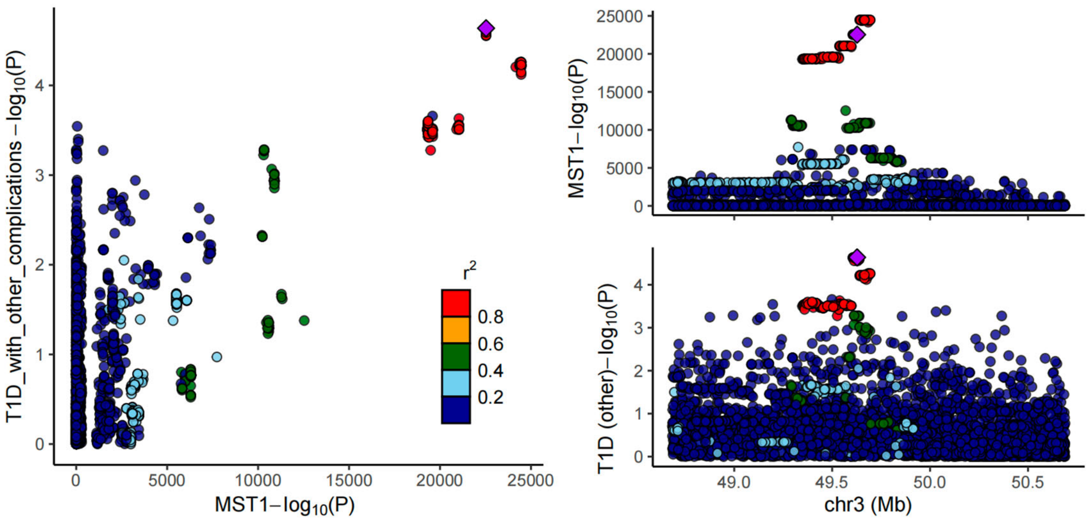

Figure S21 Regional association plots of colocalization analysis between MST1 (deCODE) and T1D with other complications.

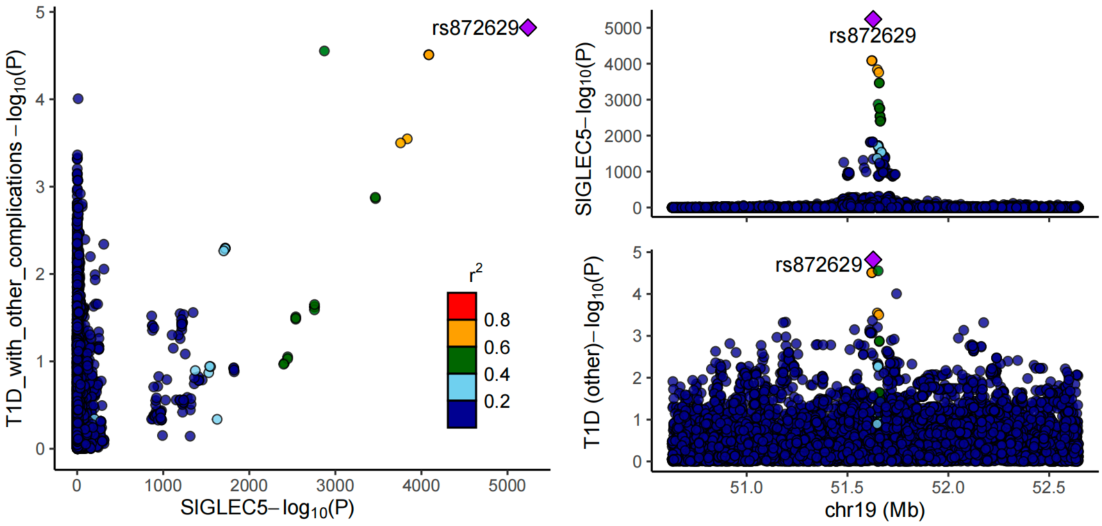

Figure S22 Regional association plots of colocalization analysis between SIGLEC5 (deCODE) and T1D with other complications.

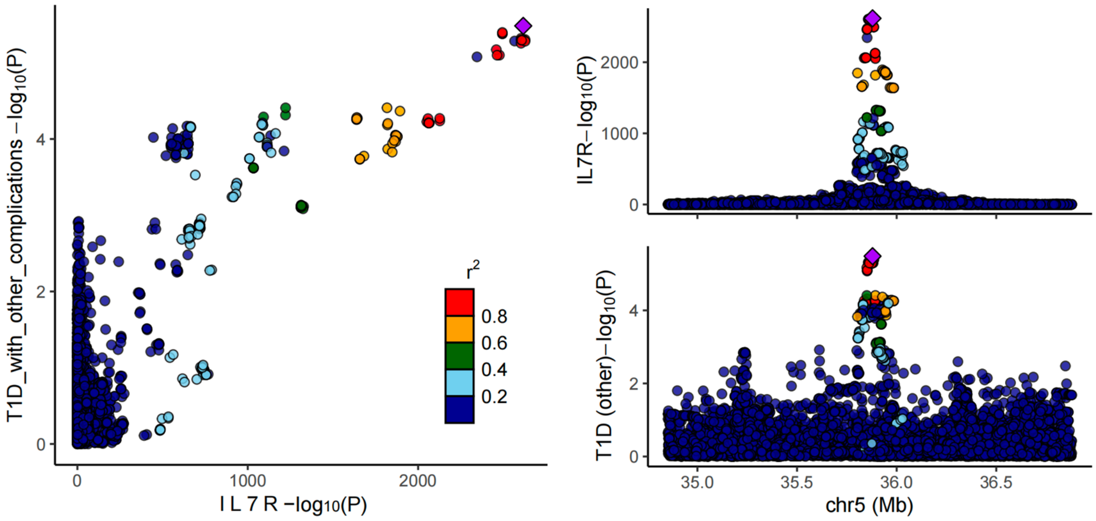

Figure S23 Regional association plots of colocalization analysis between IL7R (UKBPPP) and T1D with other complications.

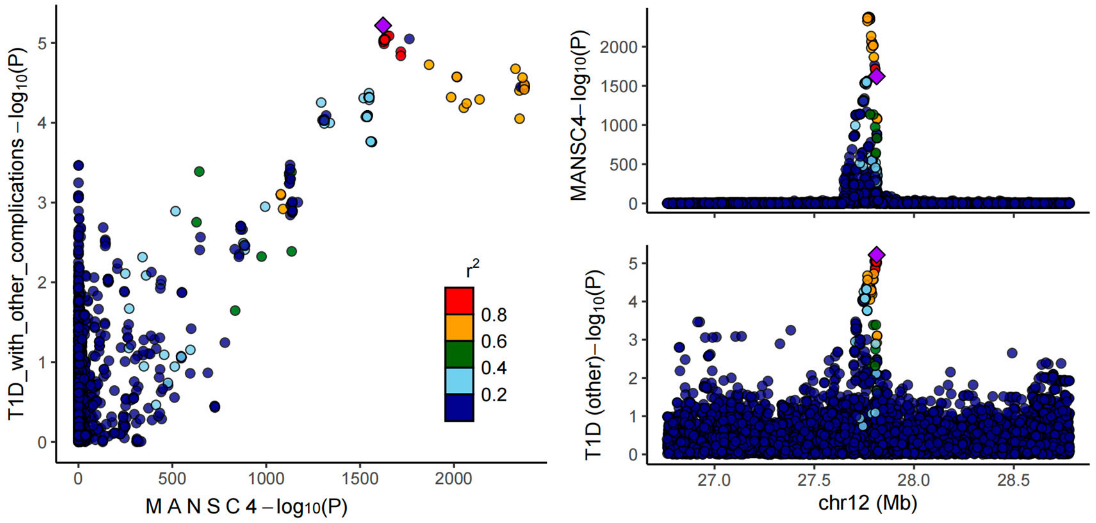

Figure S24 Regional association plots of colocalization analysis between MANSC4 (UKBPPP) and T1D with other complications.

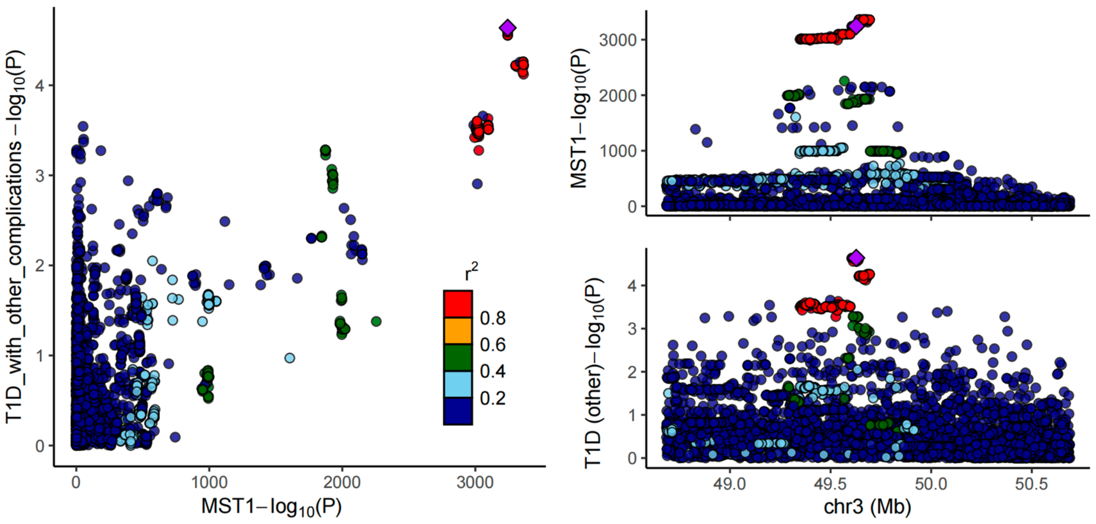

Figure S25 Regional association plots of colocalization analysis between MST1 (UKBPPP) and T1D with other complications.

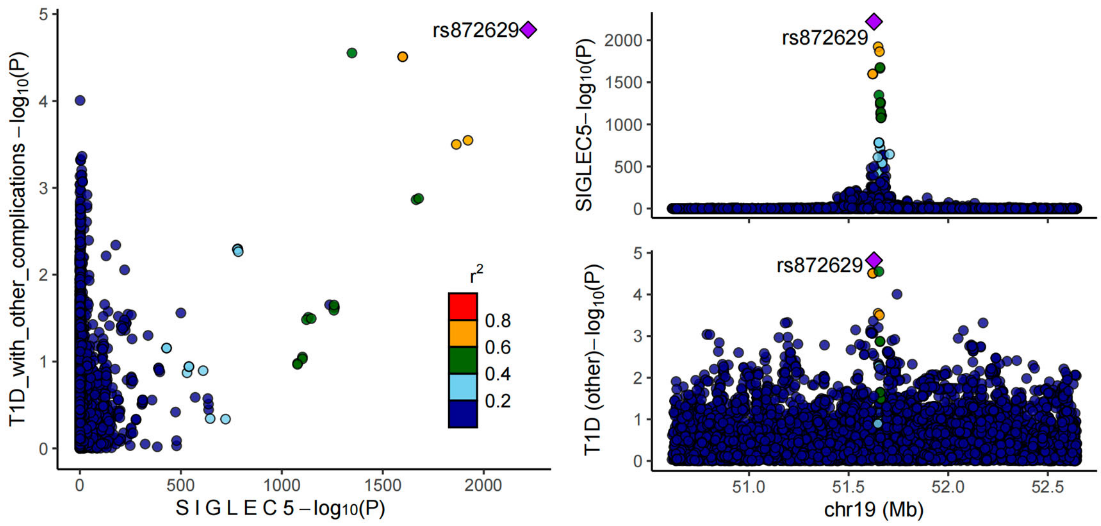

Figure S26 Regional association plots of colocalization analysis between SIGLEC5 (UKBPPP) and T1D with other complications.

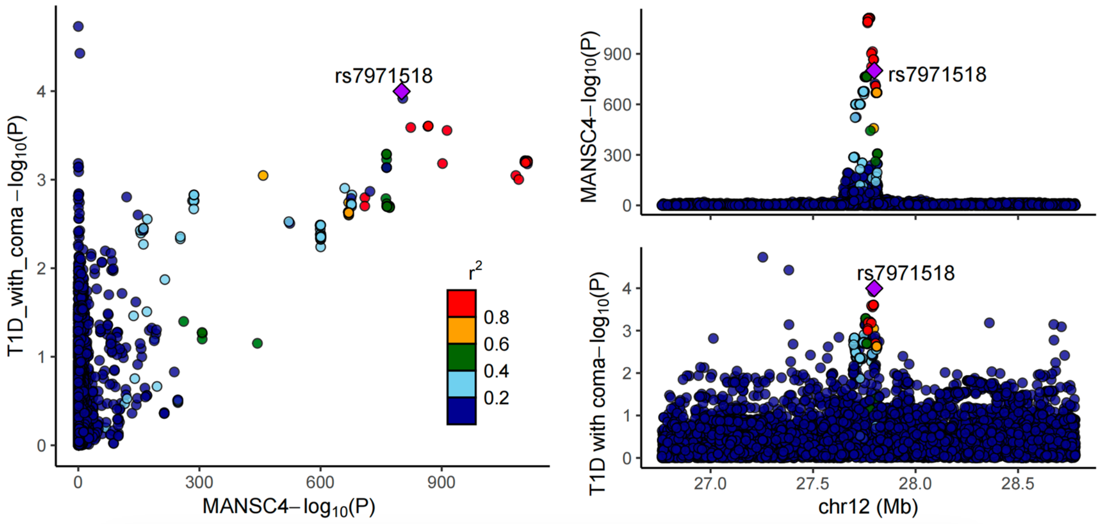

Figure S27 Regional association plots of colocalization analysis between MANSC4 (deCODE) and T1D with coma.

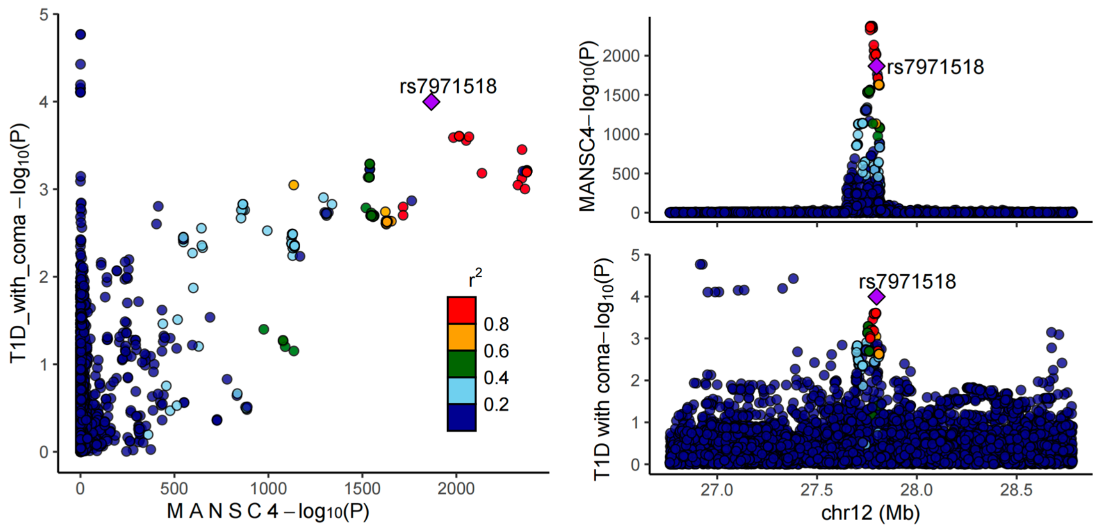

Figure S28 Regional association plots of colocalization analysis between MANSC4 (UKBPPP) and T1D with coma.

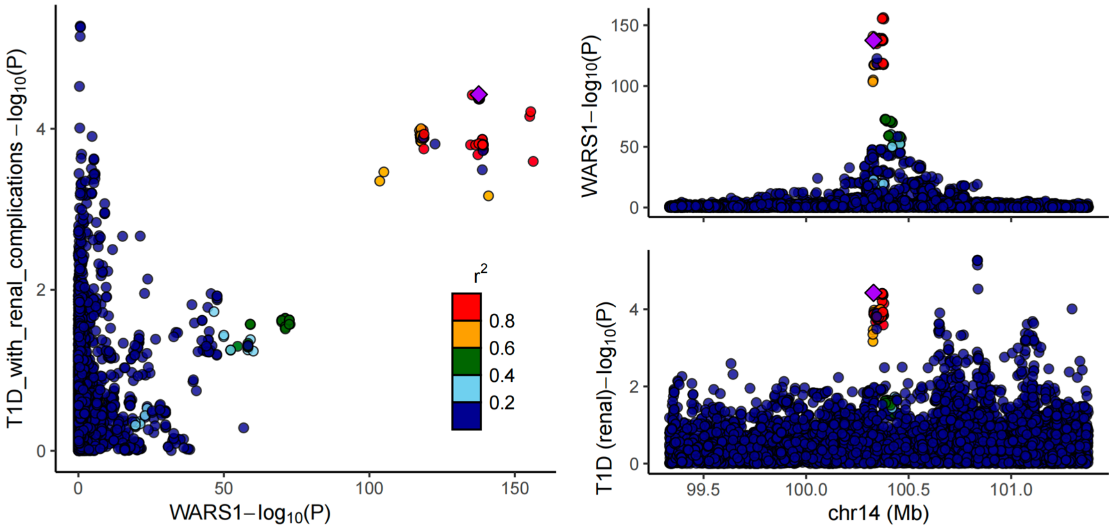

Figure S29 Regional association plots of colocalization analysis between WARS1 (UKBPPP) and T1D with renal complications.

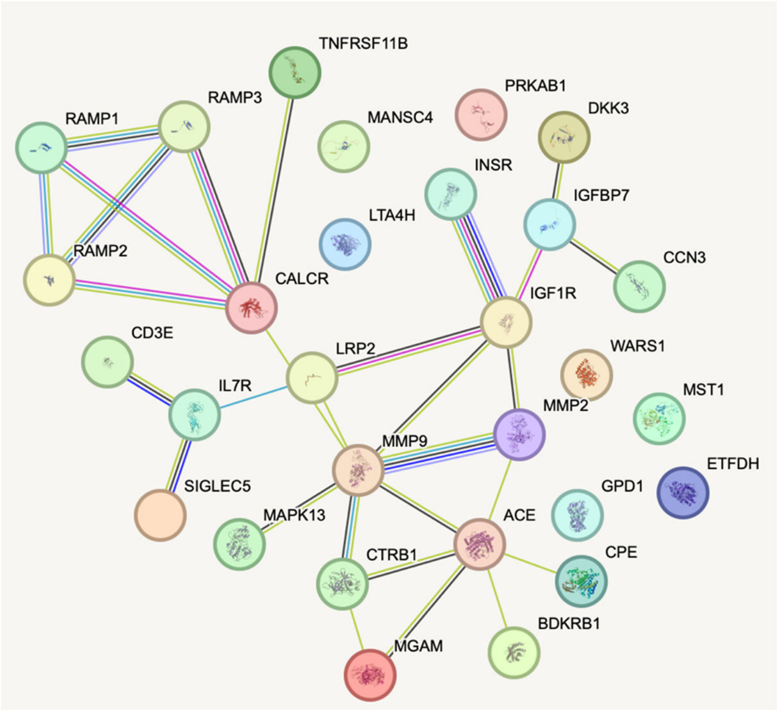

Figure S30 Protein-protein interaction (PPI) network among identified crucial protein targets and established targets of T1D.
